# Correction: Leveraging paired serology to estimate the incidence of typhoidal *Salmonella* infection in the STRATAA study

**DOI:** 10.1371/journal.pntd.0014219

**Published:** 2026-04-15

**Authors:** Jo Walker, Paula Russell, Leanne Kermack, Tan Trinh Van, Tran Vu Thieu Nga, Elli Mylona, Susana Camara, Young Chan Kim, Sonu Shrestha, Arne Gehlhaar, Josefin Bartholdson Scott, Farhana Khanam, Mila Shakya, Deus Thindwa, Melita A. Gordon, Buddha Basnyat, John D. Clemens, Firdausi Qadri, Robert S. Heyderman, Christiane Dolecek, Susan Tonks, Thomas C. Darton, Andrew J. Pollard, Stephen Baker, James E. Meiring, Merryn Voysey, Virginia E. Pitzer, and the STRATAA Study Group

There are errors in the author affiliations. The correct affiliations are as follows:

Jo Walker^1^, Paula Russell^2,3^, Leanne Kermack^2,3^, Tan Trinh Van^4^, Tran Vu Thieu Nga^4^, Elli Mylona^2,3^, Susana Camara^5^, Young Chan Kim^5^, Sonu Shrestha^5^, Arne Gehlhaar^5^, Josefin Bartholdson Scott^2,3^, Farhana Khanam^6^, Mila Shakya^7,8^, Deus Thindwa^9,10^, Melita A. Gordon^9,10,11^, Buddha Basnyat^7,12^, John D. Clemens^6,13,14,15^, Firdausi Qadri^6^, Robert S. Heyderman^9,16^, Christiane Dolecek^17^, Susan Tonks^5^, Thomas C. Darton^19^, Andrew J. Pollard^5,18^, Stephen Baker^2,3^, James E. Meiring^9,10,11^, Merryn Voysey^5,18^, Virginia E. Pitzer^1^, and the STRATAA Study Group

**1** Department of Epidemiology of Microbial Diseases, Yale School of Public Health, New Haven, Connecticut, United States of America. **2** Cambridge Institute of Therapeutic Immunology and Infectious Disease, University of Cambridge School of Clinical Medicine, Cambridge, United Kingdom. **3** Department of Medicine, University of Cambridge School of Clinical Medicine, Cambridge, United Kingdom. **4** The Hospital for Tropical Diseases, Wellcome Trust Major Overseas Programme, Oxford University Clinical Research Unit, Ho Chi Minh City, Vietnam. **5** Oxford Vaccine Group, Department of Paediatrics, University of Oxford, Oxford, United Kingdom. **6** International Centre for Diarrhoeal Disease Research, Bangladesh, Dhaka, Bangladesh. **7** Oxford University Clinical Research Unit, Patan Academy of Health Sciences, Kathmandu, Nepal. **8** Patan Academy of Health Sciences, Patan Hospital, Lalitpur, Nepal. **9** Malawi-Liverpool-Wellcome Research Programme, Blantyre, Malawi. **10** College of Medicine, University of Malawi, Blantyre, Malawi. **11** Institute of Infection, Veterinary and Ecological Sciences, University of Liverpool, Liverpool, United Kingdom. **12** Nuffield Department of Medicine, Centre for Tropical Medicine and Global Health, University of Oxford, Oxford, United Kingdom. **13** UCLA Fielding School of Public Health, University of California Los Angeles, California, United States of America. **14** International Vaccine Institute, Seoul, South Korea. **15** Korea University Vaccine Innovation Center, Seoul, South Korea. **16** Research Department of Infection, Division of Infectious Diseases, University College London, London, United Kingdom. **17** Centre for Tropical Medicine and Global Health, Nuffield Department of Medicine, University of Oxford, Oxford, United Kingdom. **18** NIHR Oxford Biomedical Research Centre, Oxford, United Kingdom. **19** Clinical Infection Research Group, School of Medicine & Population Health, University of Sheffield, Sheffield, United Kingdom.
