# Supplementary figures and images for: Leveraging paired serology to estimate the incidence of typhoidal Salmonella infection in the STRATAA study
Source: PLoS Negl Trop Dis. 2025 Oct 9;19(10):e0013612. doi: 10.1371/journal.pntd.0013612 (PMC12527133; doi:10.1371/journal.pntd.0013612)

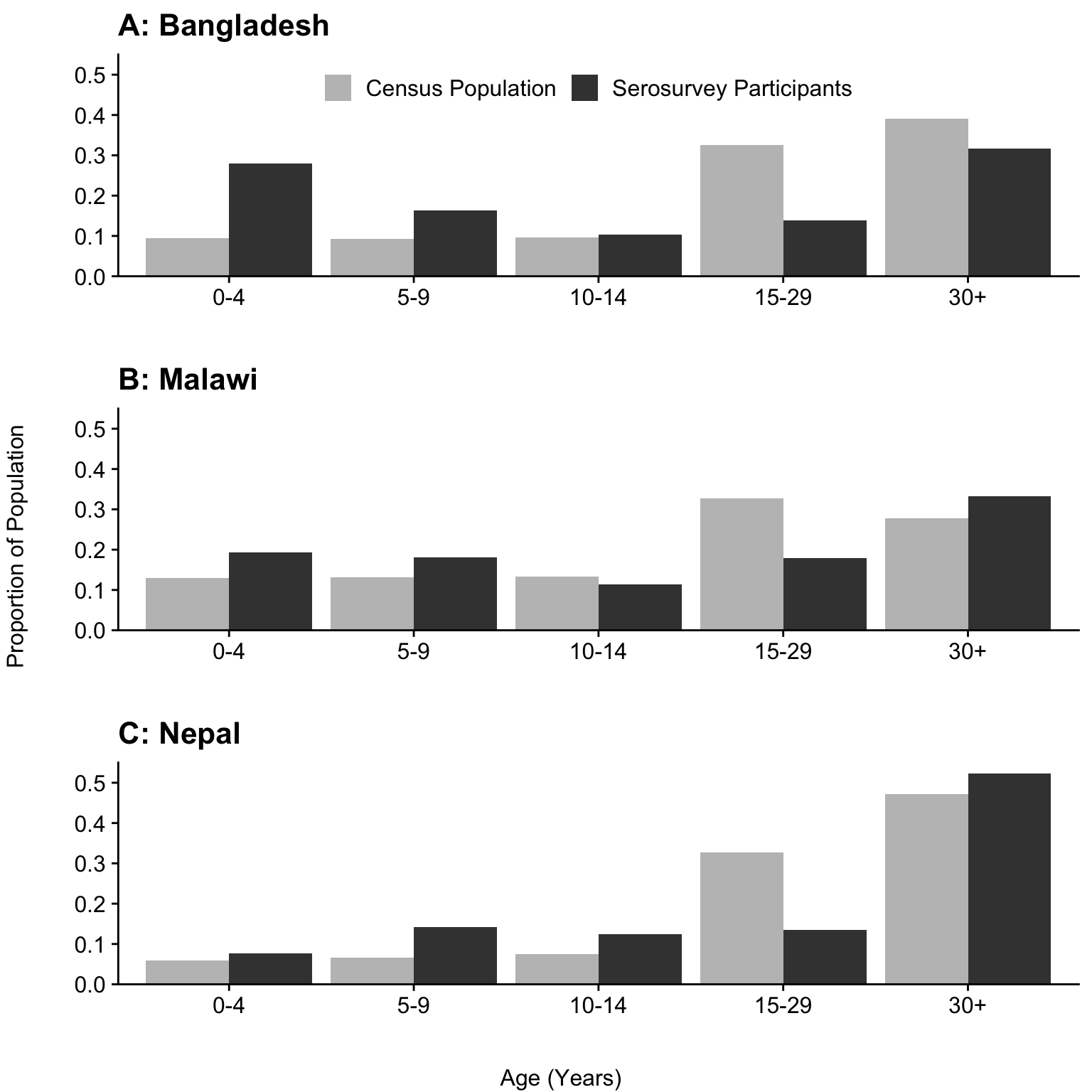

Supplement: S1 Fig — The height of each bar indicates the proportion of serosurvey participants (light grey, left) and the baseline census population (dark grey, right) in each age group for each study site. (PNG) [file pntd.0013612.s001.png]

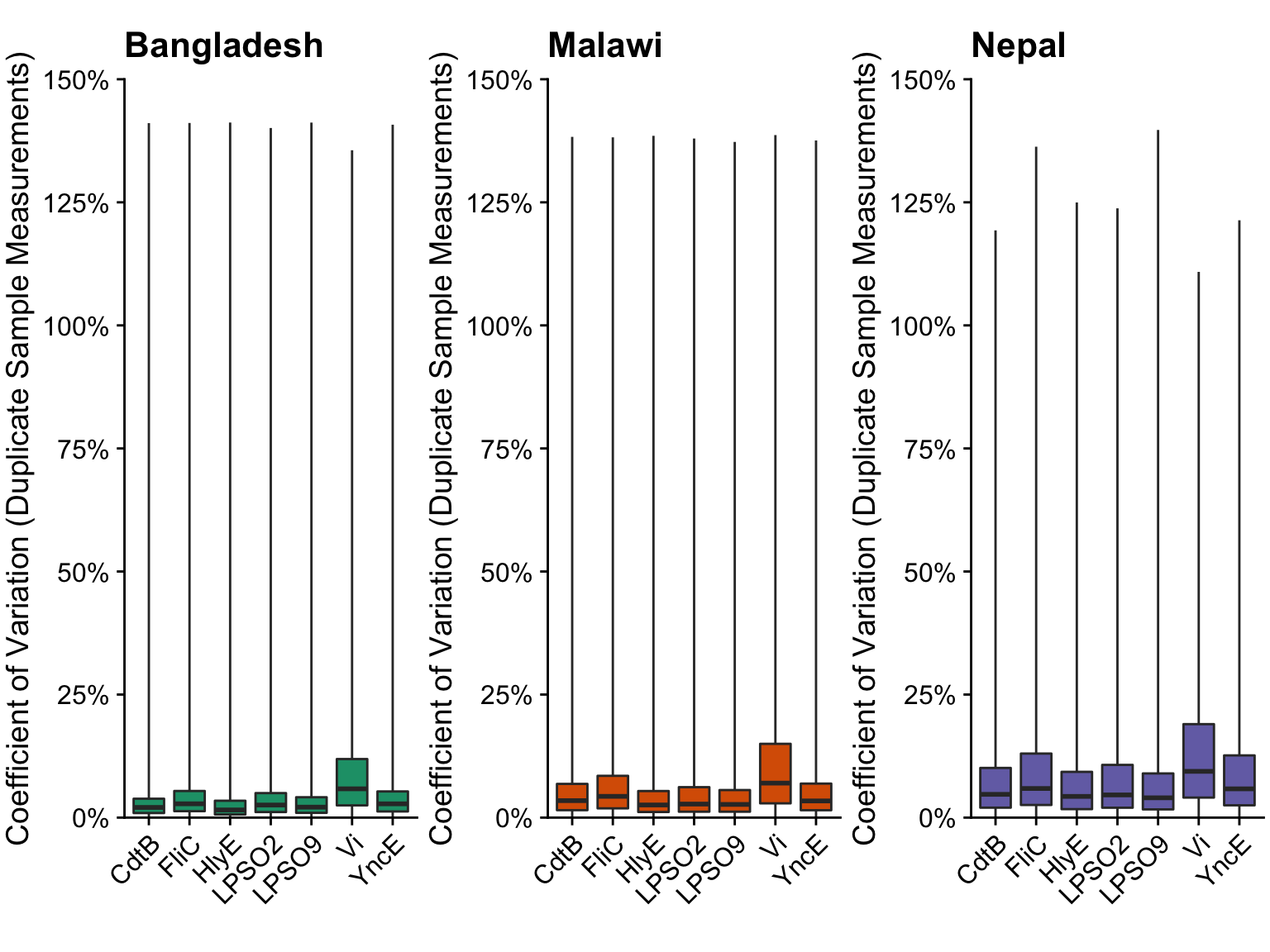

Supplement: S2 Fig — Boxplots summarize the distribution of participant’s coefficient of variation (CV) values (y-axis) for duplicate fluorescence intensity (FI) measurements. Each box corresponds to a different antigen target (x-axis), while each panel corresponds to a different study site. Box whiskers extend from the minimum to the maximum CV values. (PNG) [file pntd.0013612.s002.png]

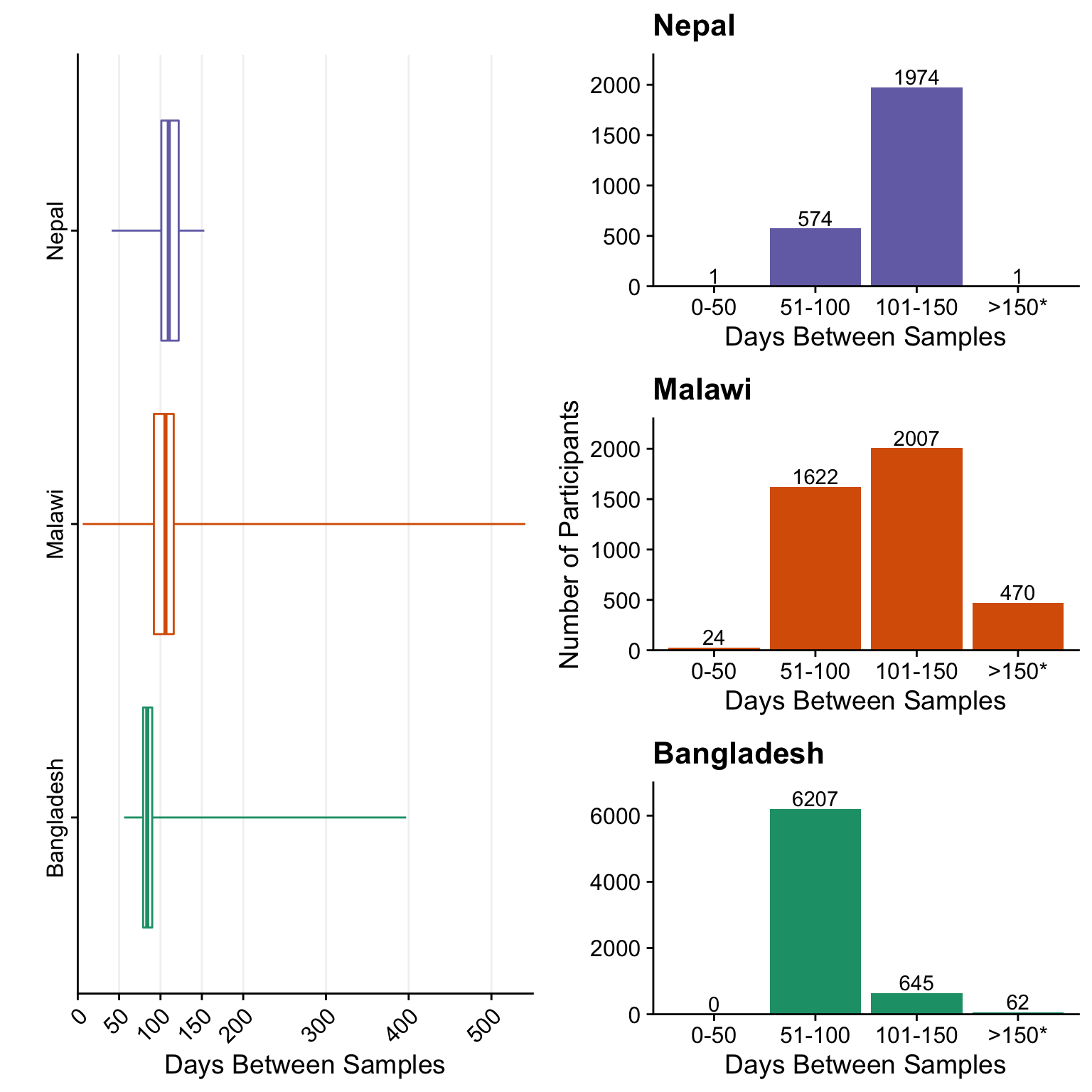

Supplement: S3 Fig — Left: Boxplots of the time between the collection of baseline and follow-up samples for participants at the Bangladesh, Malawi, and Nepal study sites. Box whiskers extend from the minimum to the maximum value. Right: Histograms of the number of participants at each study site with 0–50, 51–100, 101–150, and >150 days between samples at the Nepal (top), Malawi (middle), and Bangladesh (bottom) study sites. *Participants with >150 days between samples were excluded from the primary analysis. (PNG) [file pntd.0013612.s003.png]

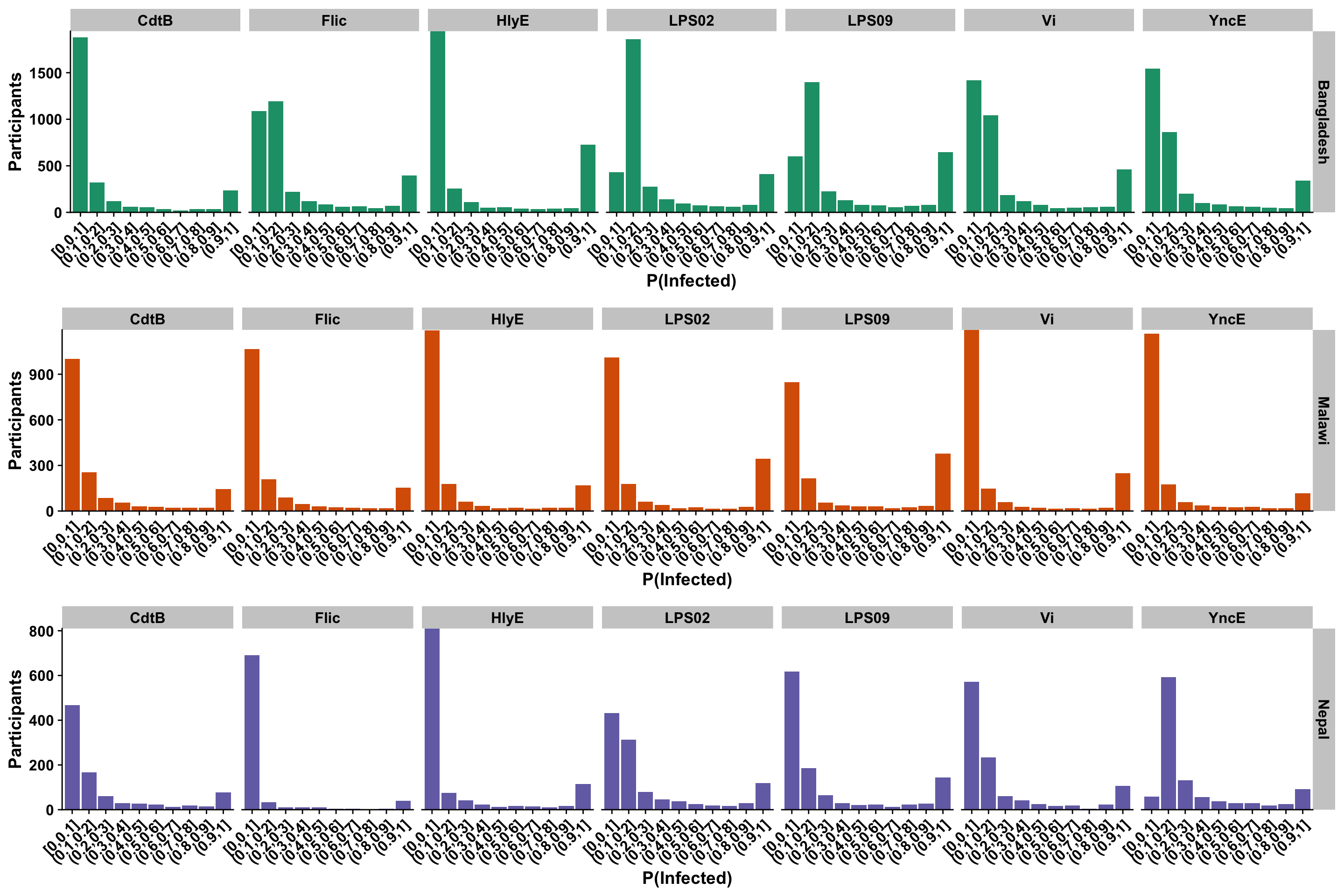

Supplement: S4 Fig — Each panel is a histogram of the posterior probability of infection (as indicated by a large rise in IgG) for participants who experienced an increase in IgG between the baseline and follow-up visits. Participants who experienced a decrease in IgG between visits were assumed to have not been infected and are not included in this figure. Each column of panels corresponds to a different antigen target, while the top, middle, and bottom rows correspond to the Bangladesh, Malawi, and Nepal study sites. (PNG) [file pntd.0013612.s004.png]

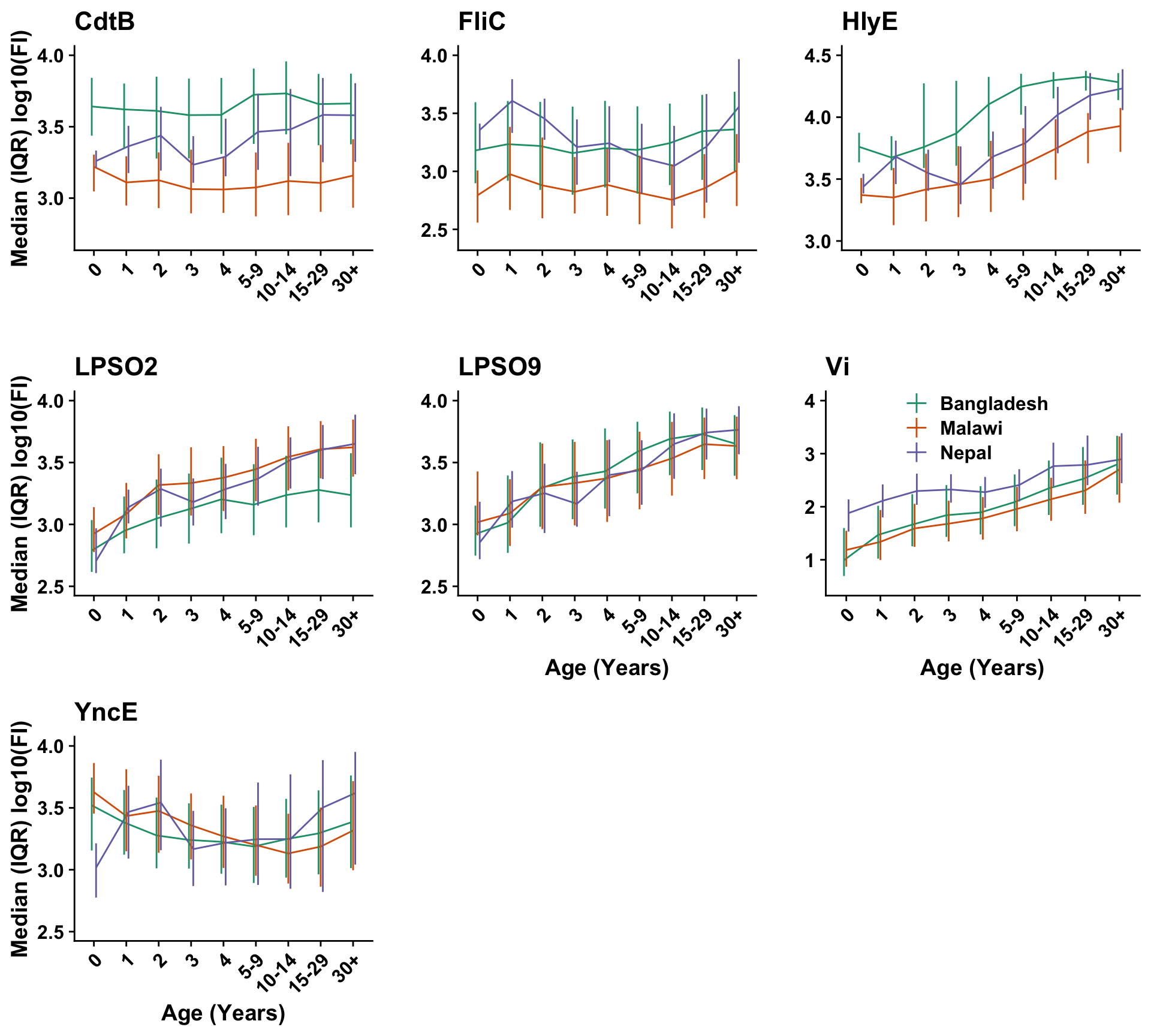

Supplement: S5 Fig — Each panel corresponds to a specific antigen target against which IgG antibodies were measured. Solid lines denote the median of the log10-transformed fluorescence intensity (FI, a proxy for IgG concentration, y-axis) across participant’s baseline samples in each age group (x-axis). Vertical lines represent the interquartile range (IQR) of the log10(FI) measurements. Green, orange, and purple lines correspond to the Bangladesh, Malawi, and Nepal study sites, respectively. (PNG) [file pntd.0013612.s005.png]

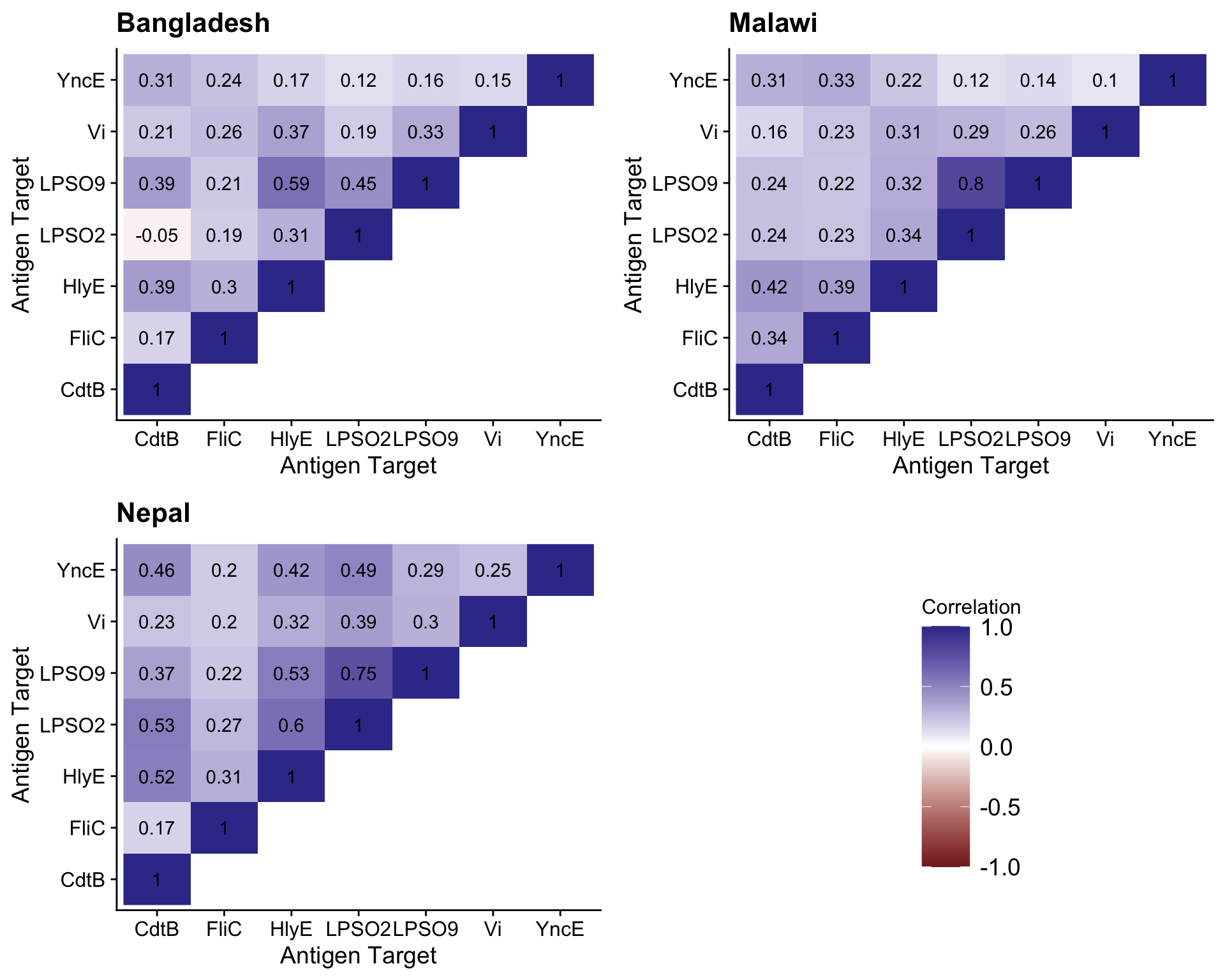

Supplement: S6 Fig — This figure displays the Pearson’s correlation coefficient of standardized IgG measurements from the same participant sample for each unique pair of antigen targets. (PNG) [file pntd.0013612.s006.png]

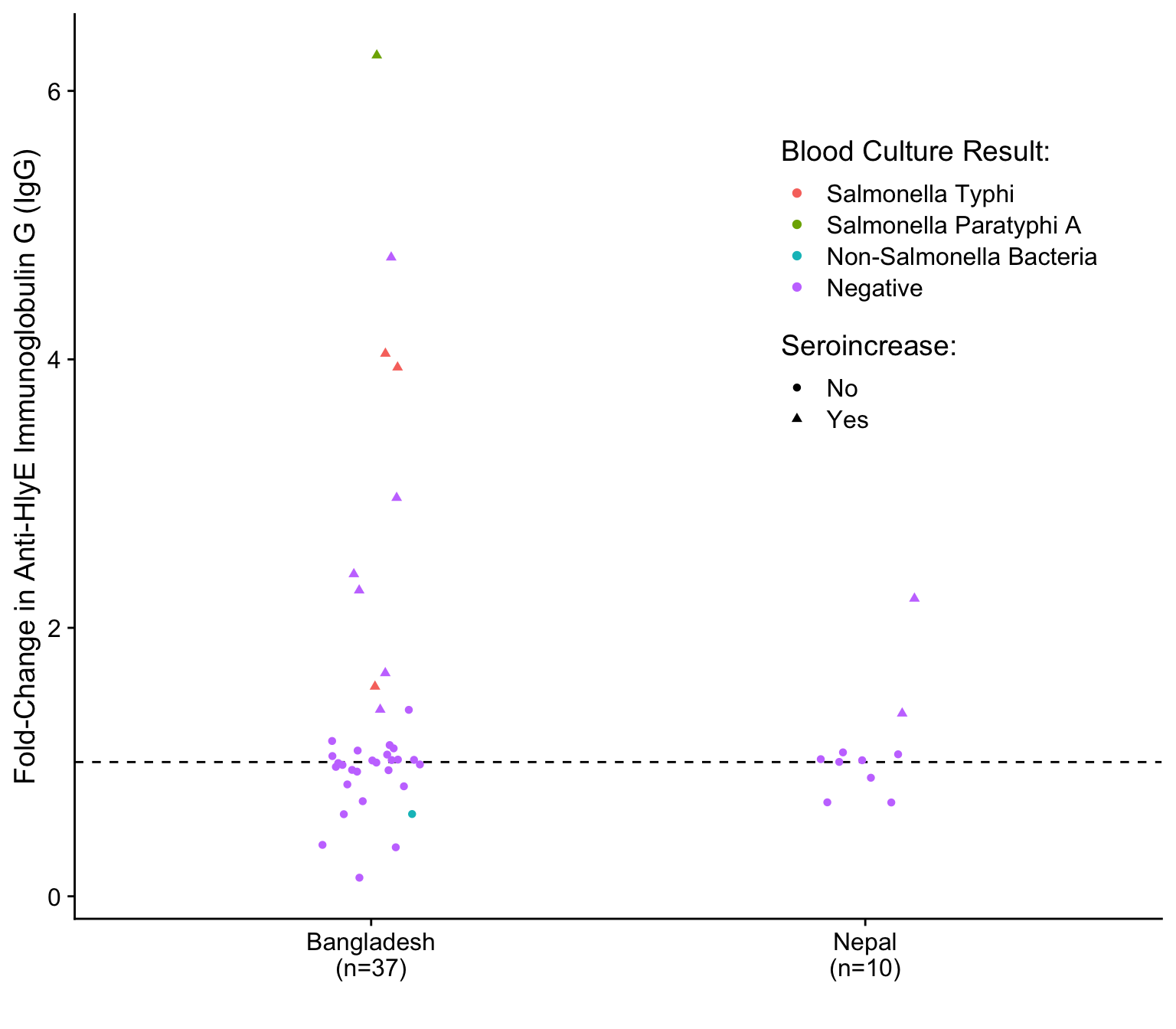

Supplement: S7 Fig — Each point represents a single serosurvey participant who also had a blood-culture test performed at a STRATAA study clinic during the period between their two serologic samples. The y-axis indicates the fold-change in anti-HlyE IgG between the baseline and follow-up sample. Participants classified as infected by the mixture model on the basis of their HlyE seroresponse are plotted as triangles. All other participants are plotted as circles. The color of each point indicates the blood-culture test result. Individual points have been adjusted horizontally to prevent visual overlap. (PNG) [file pntd.0013612.s007.png]

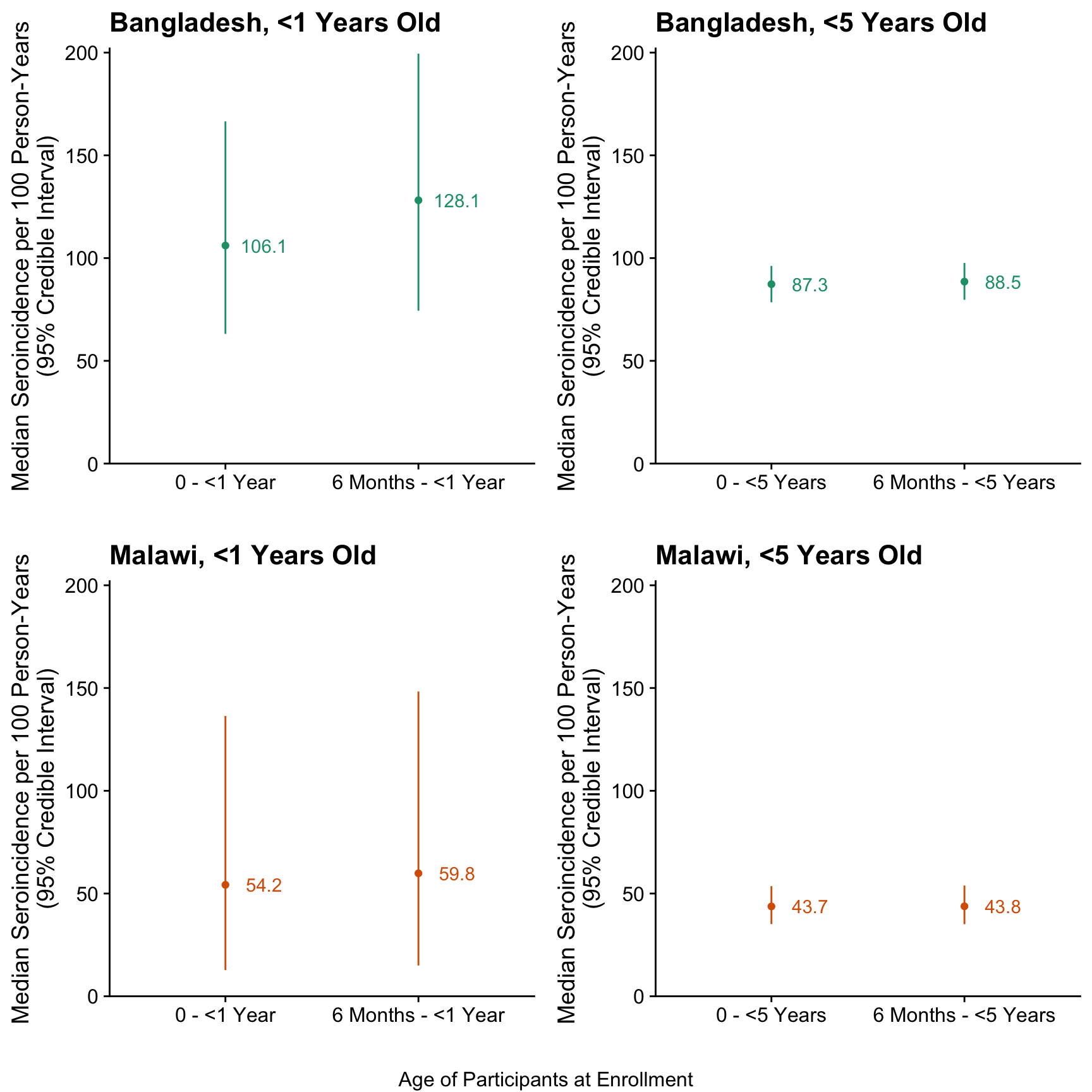

Supplement: S8 Fig — Points and lines represent the median estimate and 95% credible interval of HlyE seroincidence, respectively. Panels in the top row correspond to the Bangladesh study site, while those in the bottom row correspond to the Malawi site. Panels in the left column are based on participants who were <1 year old at the time of enrollment, while panels in the right column are based on participants who were <5 years old at enrollment. Within each panel, the values on the left and right represent the seroincidence estimate when participants <6 months old are included and excluded, respectively. No participants <6 months of age were enrolled at the Nepal site, so it is not included in this figure. (PNG) [file pntd.0013612.s008.png]

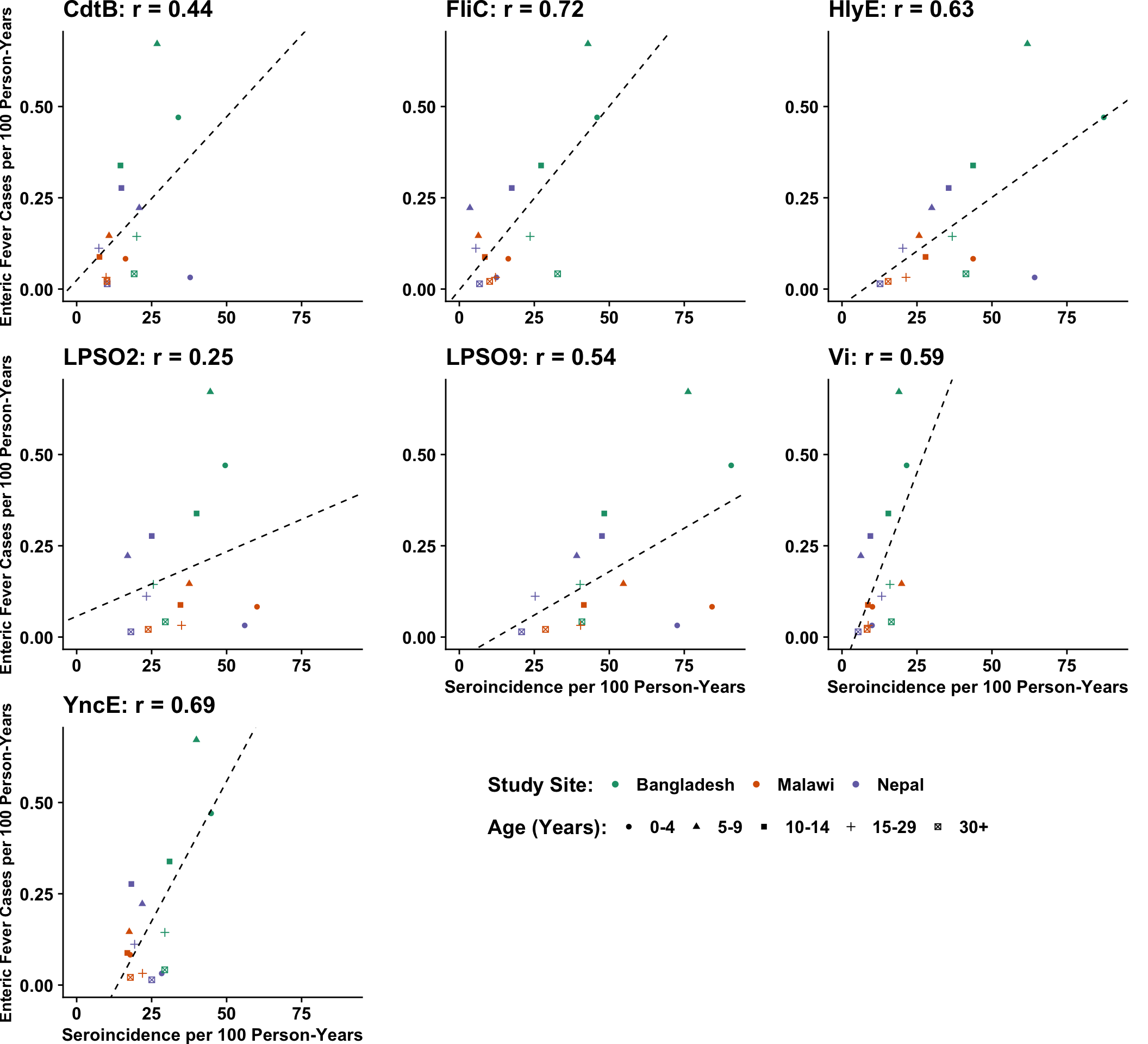

Supplement: S9 Fig — Each panel corresponds to the specific antigen target which was used to classify participants’ infection status when calculating seroincidence. Each point corresponds to an age group (shape) at a given study site (color). The position of each point represents the seroincidence (x-axis) and unadjusted enteric fever incidence (y-axis) for that age group and study site during the study period. The linear relationship between seroincidence and enteric fever incidence is indicated by a dashed line of best fit and the Pearson’s correlation coefficient (r) at the top of the panel. (PNG) [file pntd.0013612.s009.png]

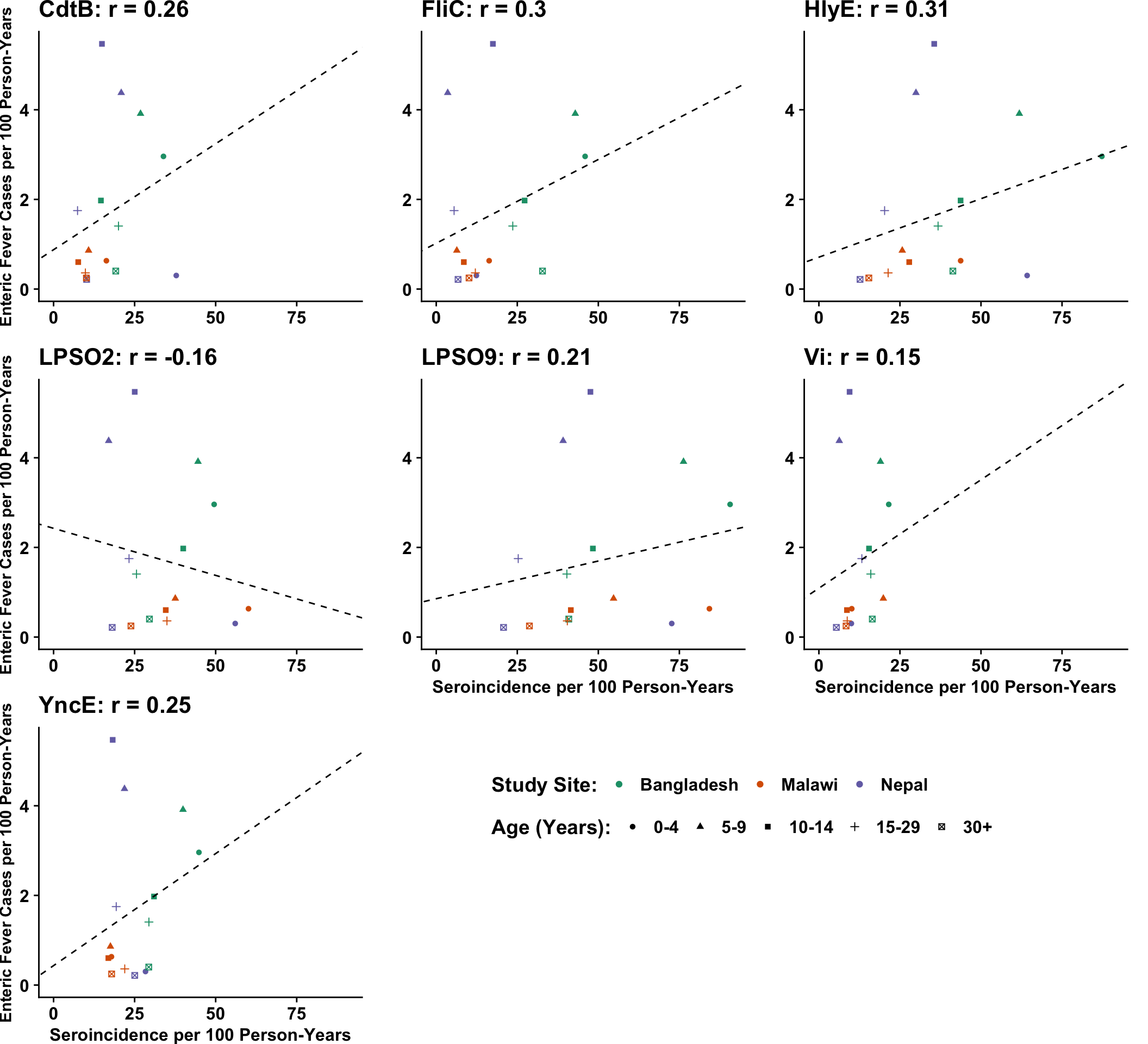

Supplement: S10 Fig — Each panel corresponds to the specific antigen target which was used to classify participants’ infection status when calculating seroincidence. Each point corresponds to an age group (shape) at a given study site (color). The position of each point represents the seroincidence (x-axis) and adjusted enteric fever incidence (y-axis) for that age group and study site during the study period. The linear relationship between seroincidence and enteric fever incidence is indicated by a dashed line of best fit and the Pearson’s correlation coefficient (r) at the top of the panel. (PNG) [file pntd.0013612.s010.png]

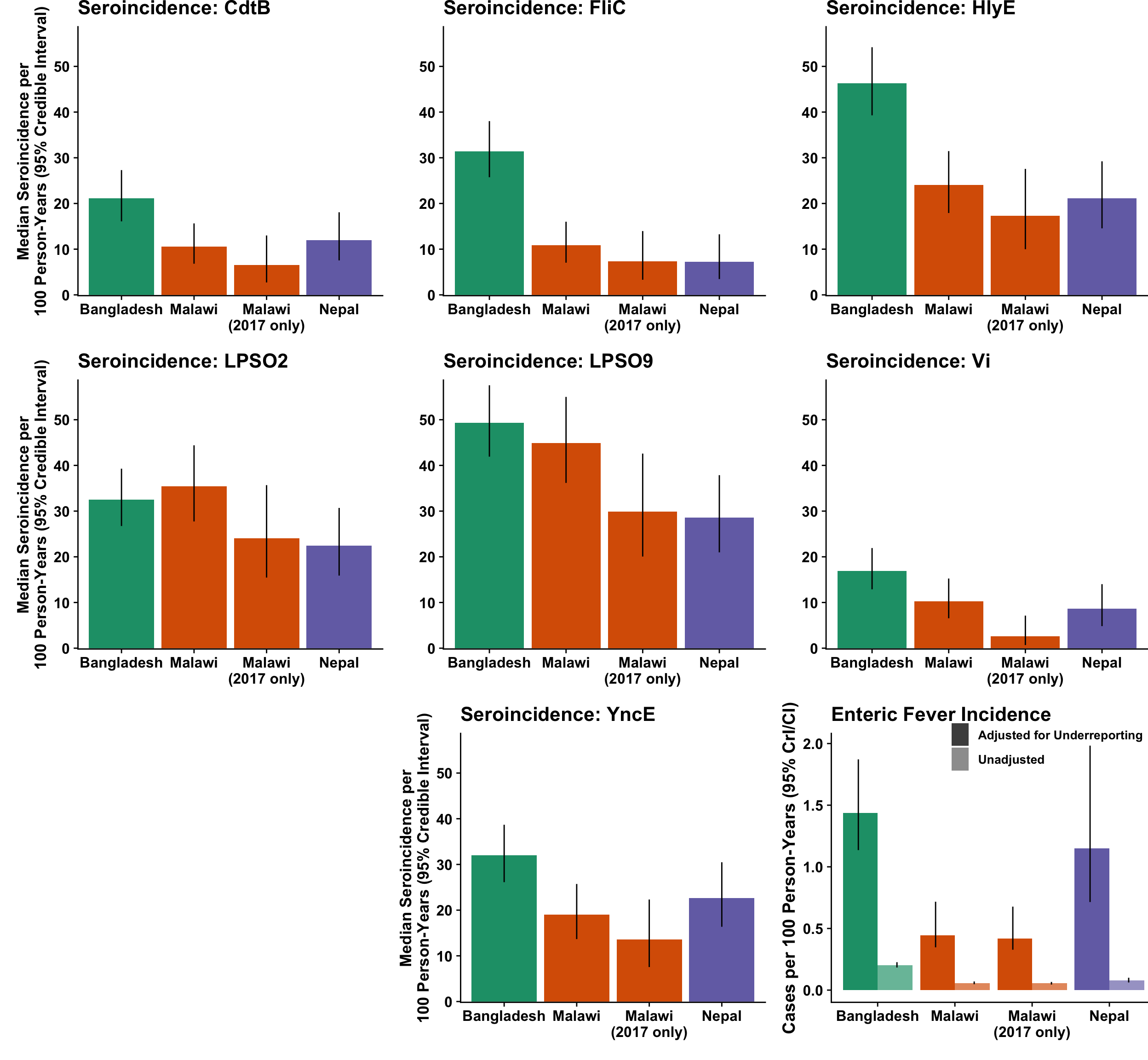

Supplement: S11 Fig — Each bar corresponds to a different study site, and the bar heights represent either the seroincidence of infection or the incidence of enteric fever cases at each site, as indicated. Error bars represent the 95% credible interval (CrI) for the estimates of seroincidence and adjusted enteric fever incidence, and 95% confidence intervals (CI) for unadjusted enteric fever incidence. Incidence measures in this figure correspond to the overall population at each study site and its unique age distribution, rather than any specific age stratum. Since the Malawi serosurvey overlapped with two seasonal peaks in transmission, we included a sensitivity analysis limiting the Malawi site to participants with both samples collected in 2017. (PNG) [file pntd.0013612.s011.png]

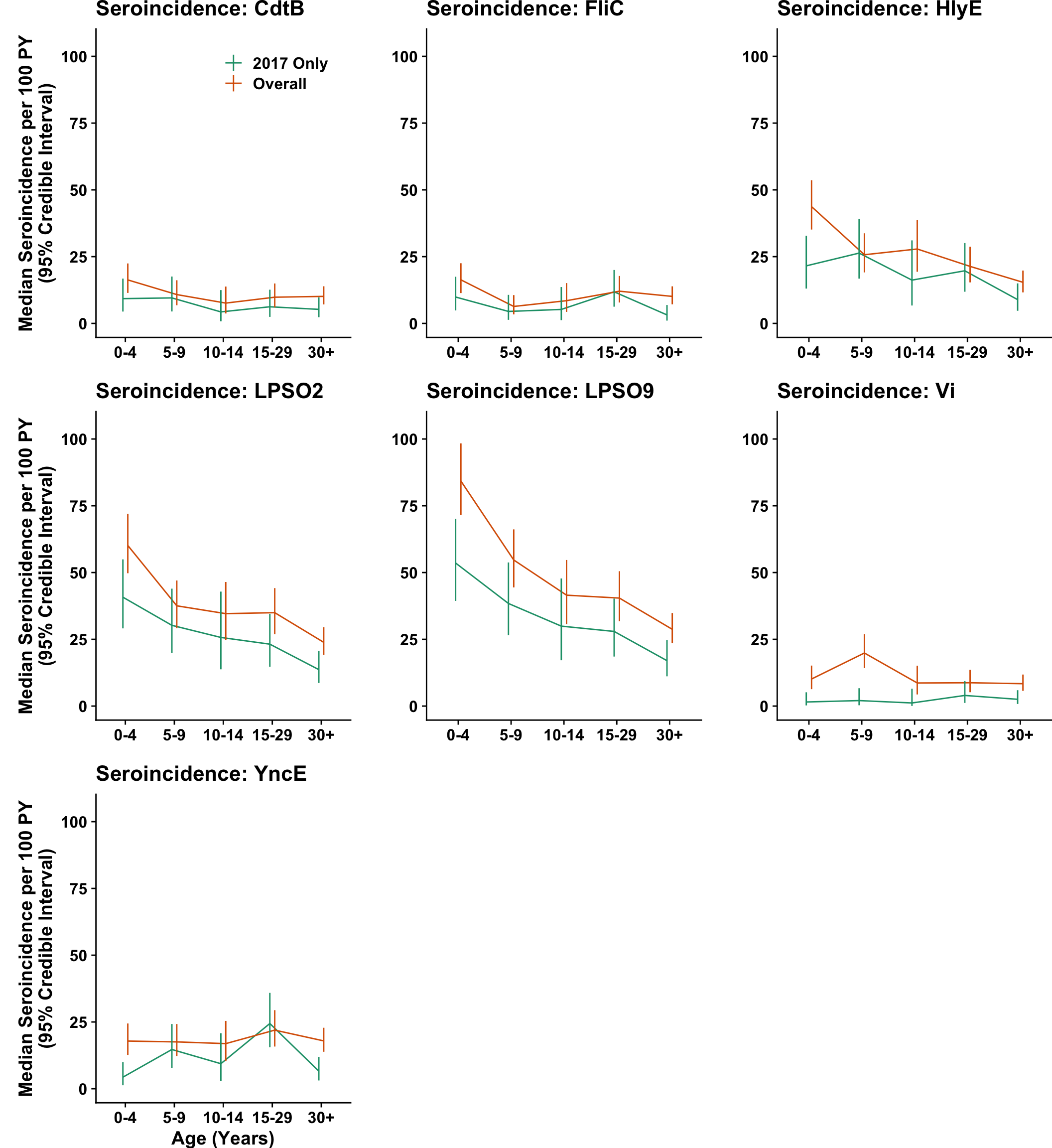

Supplement: S12 Fig — Each panel corresponds to the specific antigen target which was used to classify participants’ infection status when calculating seroincidence. Solid lines denote the median seroincidence (y-axis) in each age group (x-axis). Vertical lines represent the 95% credible intervals of the seroincidence estimates, and dashed lines indicate the adjusted incidence of enteric fever. Orange lines represent estimates based on the full serosurvey period, while green lines correspond to estimates limited to participants with both samples collected in 2017. (PNG) [file pntd.0013612.s012.png]

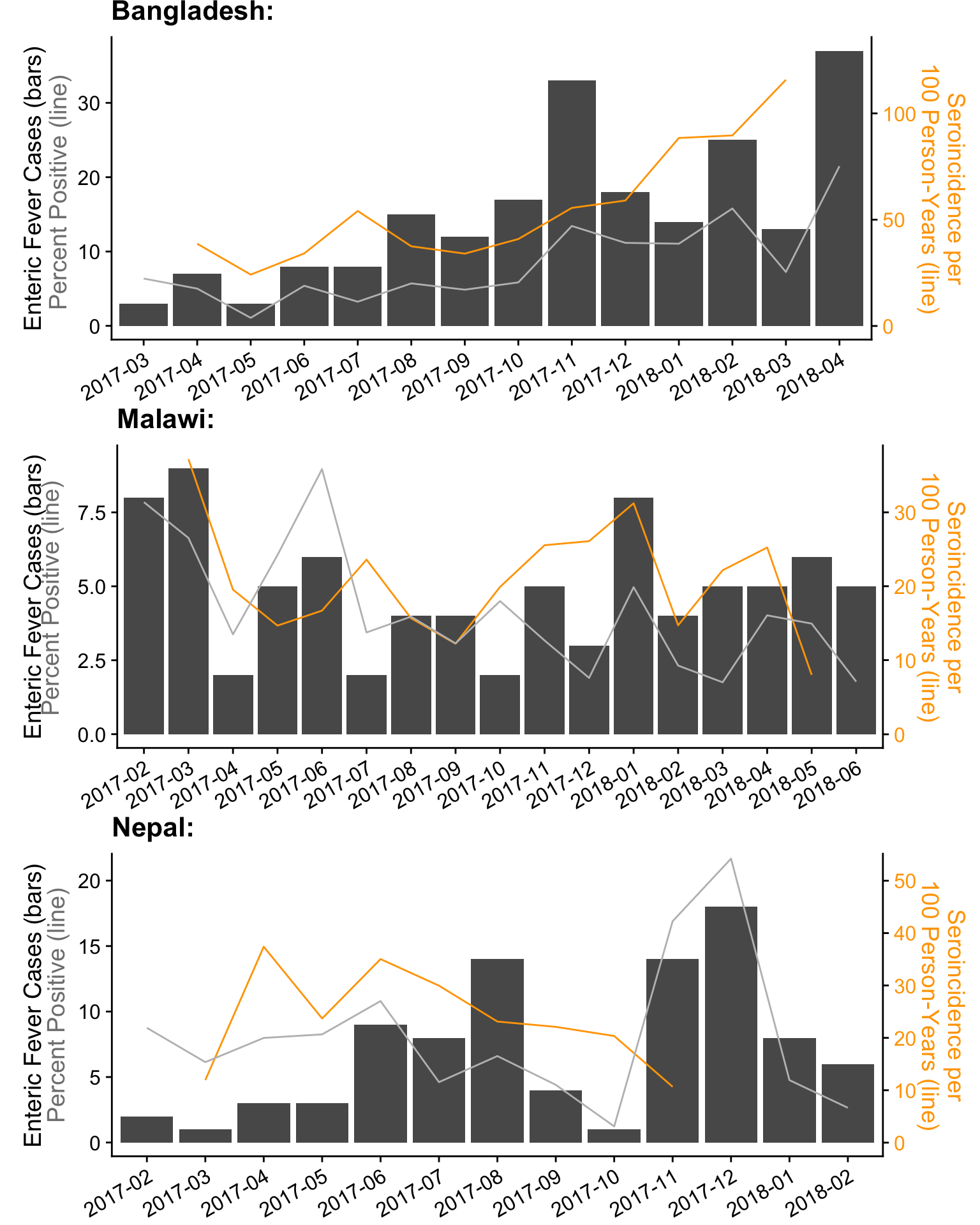

Supplement: S13 Fig — The height of each bar indicates the monthly number of blood-culture-confirmed enteric fever cases. The orange line represents the monthly estimated HlyE seroincidence, with each participant assigned to the month of the midpoint between their baseline and follow-up samples. The top, middle, and bottom panels correspond to the Bangladesh, Malawi, and Nepal STRATAA sites, respectively. Seroincidence estimates based on fewer than 50 participants are suppressed (April and June 2018 in Bangladesh and Malawi, respectively). (PNG) [file pntd.0013612.s013.png]

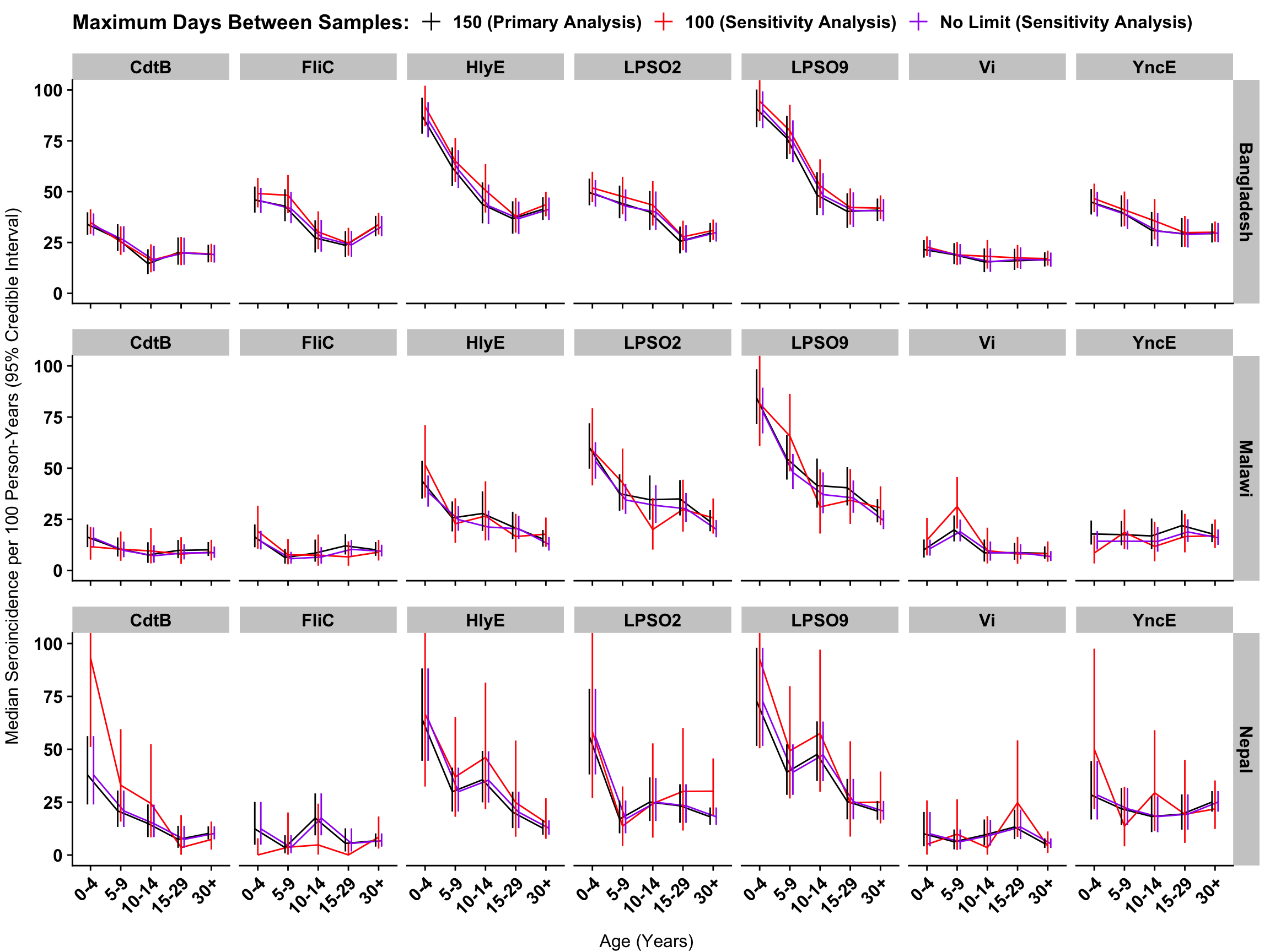

Supplement: S14 Fig — Each panel corresponds to the specific antigen target which was used to classify participants’ infection status when calculating seroincidence. Seroincidence estimates for the Bangladesh, Malawi, and Nepal study sites appear in the top, middle, and bottom row of panels, respectively. Solid lines denote the median seroincidence (y-axis) in each age group (x-axis). Vertical lines represent the 95% credible intervals of the seroincidence estimates. Black lines correspond to the primary analysis, which excluded participants with sample pairs collected over 150 days apart, while red lines correspond to a sensitivity analysis which only included participants with < 100 days between samples. (PNG) [file pntd.0013612.s014.png]

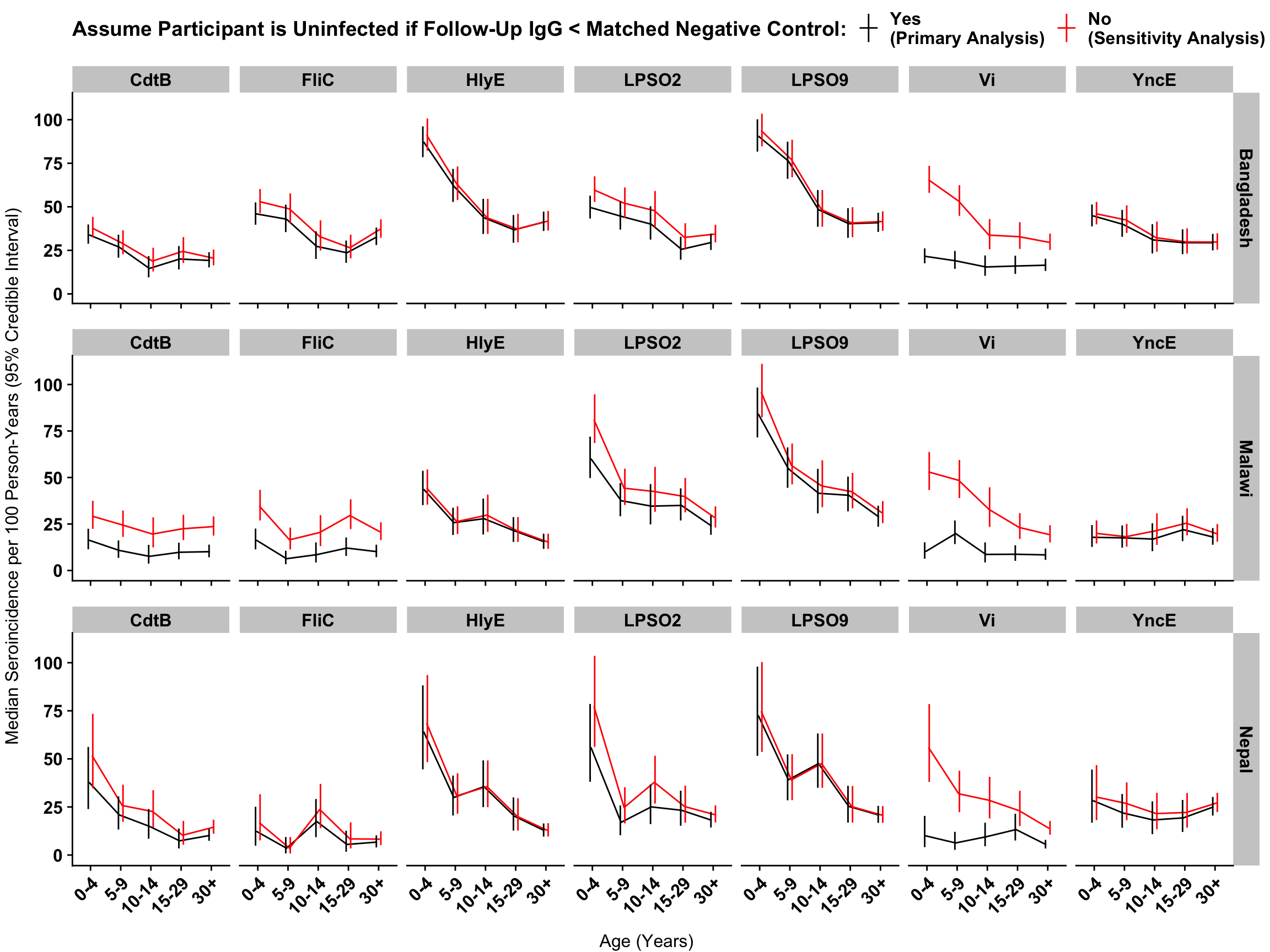

Supplement: S15 Fig — Each panel corresponds to the specific antigen target that was used to classify participants’ infection status when calculating seroincidence. Seroincidence estimates for the Bangladesh, Malawi, and Nepal study sites appear in the top, middle, and bottom row of panels, respectively. Solid lines denote the median seroincidence (y-axis) in each age group (x-axis). Vertical lines represent the 95% credible intervals of the seroincidence estimates. Black lines correspond to the primary analysis, while red lines correspond to a sensitivity analysis in which participants with a lower IgG at follow-up than a plate- and antigen batch-specific negative control are automatically classified as uninfected, regardless of the relative change in IgG between samples. (PNG) [file pntd.0013612.s015.png]

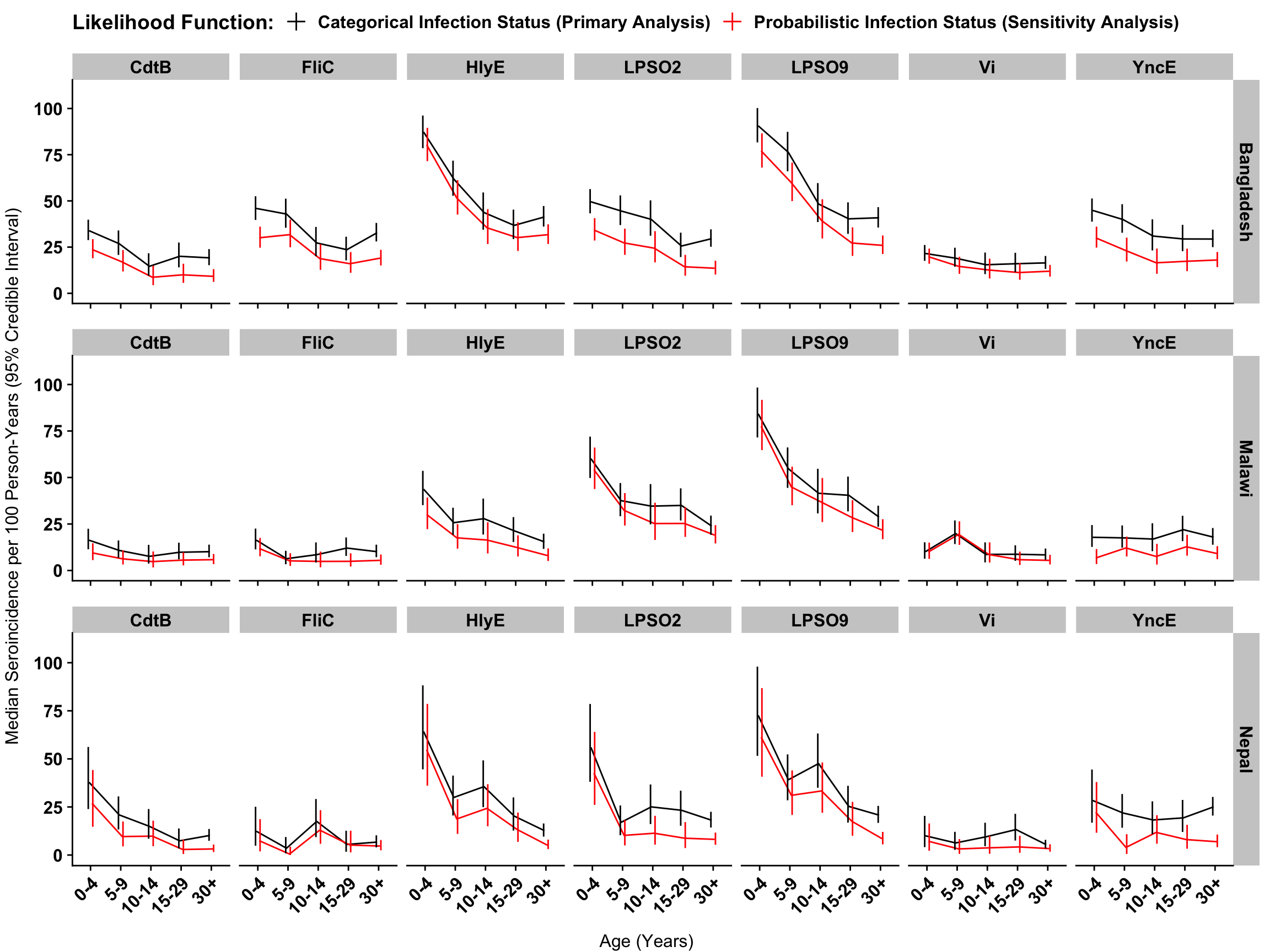

Supplement: S16 Fig — Each panel corresponds to the specific antigen target which was used to classify participants’ infection status when calculating seroincidence. Seroincidence estimates for the Bangladesh, Malawi, and Nepal study sites appear in the top, middle, and bottom row of panels, respectively. Solid lines denote the median seroincidence (y-axis) in each age group (x-axis). Vertical lines represent the 95% credible intervals of the seroincidence estimates. Black lines correspond to the primary analysis, while red lines correspond to a sensitivity analysis in which the likelihood function for seroincidence directly incorporates participants’ mixture model-derived probability of infection. (PNG) [file pntd.0013612.s016.png]

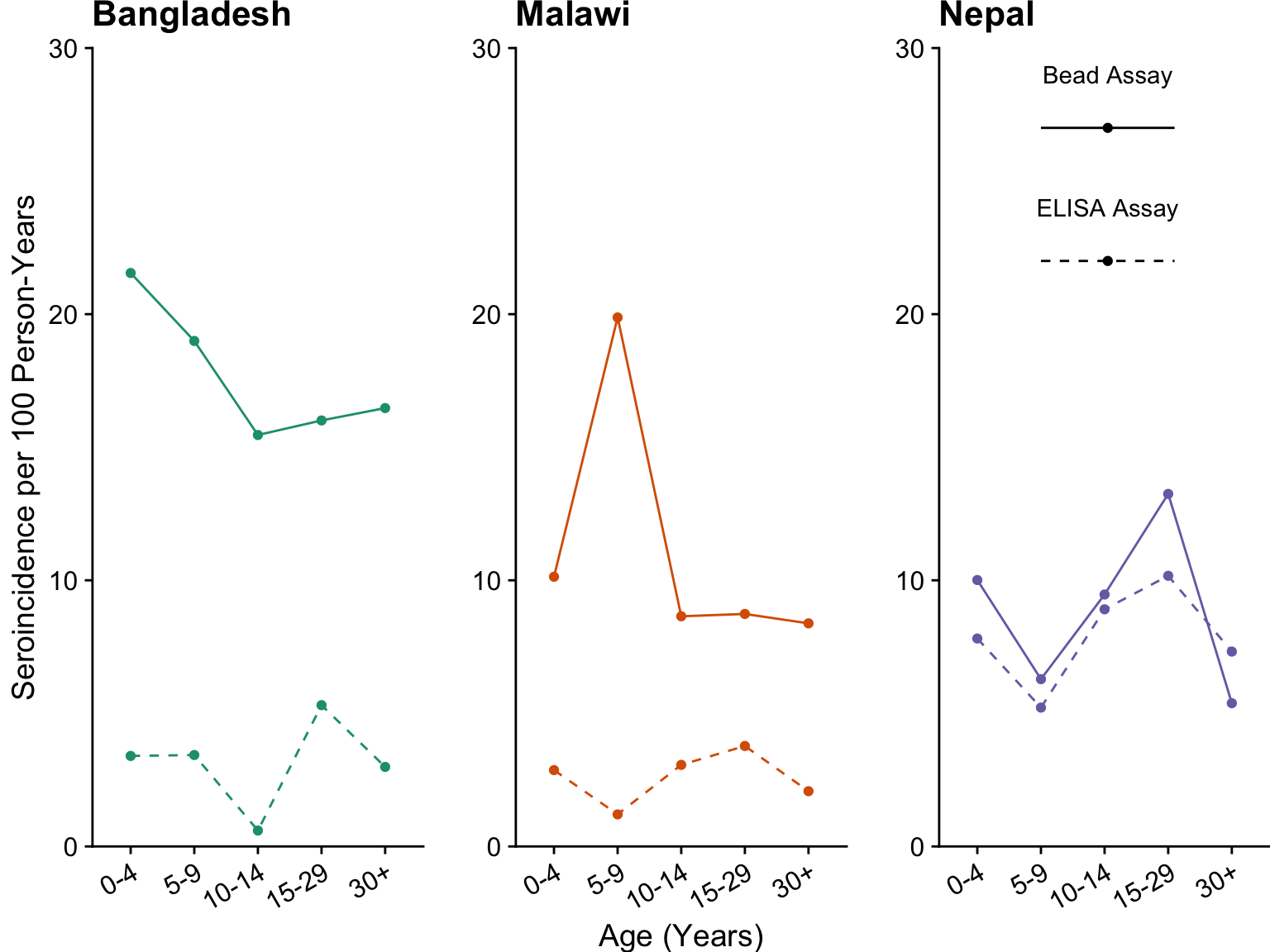

Supplement: S17 Fig — Each panel corresponds to a different study site. Lines denote the estimated seroincidence (y-axis) in each age group (x-axis). Solid lines represent seroincidence estimates based on the serologic data generated in this study with a bead-based multiplex assay, while the dashed lines depict previously published seroincidence estimates based on ELISA assays. Green, orange, and purple lines correspond to the Bangladesh, Malawi, and Nepal study sites, respectively. (PNG) [file pntd.0013612.s017.png]
