# Supplementary material for: Leveraging paired serology to estimate the incidence of typhoidal Salmonella infection in the STRATAA study
Source: PLoS Negl Trop Dis. 2025 Oct 9;19(10):e0013612. doi: 10.1371/journal.pntd.0013612 (PMC12527133; doi:10.1371/journal.pntd.0013612)
Supplement: Attachment — Submitted filename: typhoid_seroincidence_review.docx [file pntd.0013612.s018.docx]

In “Leveraging paired serology to estimate the incidence of typhoidal *Salmonella* infection in the STRATAA study,” the authors use paired blood samples to estimate the seroincidence of typhoid incidence across multiple countries, age groups, and antigen targets. The authors then compare these estimates to adjusted and unadjusted blood-culture-confirmed incidence estimates. This method and these findings fill a much-needed gap in typhoid burden estimates, linking seroincidence to symptomatic healthcare-seeking cases. The study is well-written and the method and findings are robust, and I have mostly minor comments and suggestions and some requests for further justification.

Minor note—there are no page numbers or line numbers, so I noted my comments by sections and paragraphs

**Introduction:**

- Suggestion for wording in Paragraph 1 of introduction: “As a result, enteric fever mostly occurs in settings with limited [**access to**] clean water and sanitation…”
- Suggestion for wording in paragraph 2 of introduction: “The absence of [**robust and routine**] surveillance data [**limits evidence-based decision making regarding typhoid control and prevention without complete data**]”
- The third paragraph in the introduction needs some sort of transition; as it is, it seems a little abrupt and out of the blue. It would be helpful to start with a transition sentence offering serosurveillance as a solution to the lack of routine surveillance data.
- The last paragraph of the introduction does not make the objective of the study as clear as it should be (as it is in the abstract). In that paragraph, it would be helpful to see: 1) motivation and rationale for the study, 2) (optional) brief overview of methods, and 3) and a reframing of the objective of the study. This is unclear as it currently is.

**Methods:**

- In the first paragraph of the methods (Study design and enrollment of participants), were there no census updates in Malawi? To clarify, there were baseline censuses and final censuses at each site, and Nepal and Bangladesh had additional census updates in between? Please make this clearer.
- In “Sample collection and laboratory testing,” please provide the percentage of children younger than 6 months old who were included. Can you provide the distribution of ages sampled? Was this age group oversampled? Was sampling done in proportion to age distribution of the population? Also, please rephrase “participants who could be located.” Can the authors provide reasons for lost follow up (apart from not being able to find the participants—and why is that? Emigration?)?
- Please provide justification for choosing to use the mixture model to address the study question. Why use this model over, for example, an exponential decay model?
- There are often issues with convergence in mixture models. Please provide further model diagnostics for convergence past visual inspection.
- Why did the authors use the Metropolis algorithm over Metropolis-Hastings?
- Please cite R in the text (last paragraph before “Ethics”).
- In the first paragraph of the Results, please (re)state the inclusion criteria for the participants.

**Results**

- Please show the distribution of days between samples (perhaps in a graph). Are there any concerns or biases with having visits too close together? Does the fact that follow-up samples were collected slightly earlier in Bangladesh (vs. later for others) have any implications for the results?
- General comments for figures:
  - Please spell out all acronyms (IgG, CrI, PY, etc)
  - Suggest using the word “panel” instead of subfigure
- Table 2: shouldn’t the adjusted numbers be CrI (not CI)?
- Table 2: Are the ratios shown using the median estimates? Why not have uncertainty intervals for the ratios too?
- Why use 95% CrIs in Table 2, but report IQR in Table 1 and the text for other estimates?
- In Table 3, please provide further details about the table (i.e., I think this is Pearson correlation?). Why are there columns without Nepal?
- Figure 1: please label the scales in the middle column. Also, no need to capitalize X- and Y-axis (and this isn’t done in Figure 2)
- Figure 2: Can the authors put the legend altogether, instead separated by two panels? Can the authors also add the 95% CrIs for the adjusted incidence estimates?
- Figure 3 & 4: please add labels for the row panels.
- Supplementary Figures 3 & 4: (optional) consider adding a line for each site; or perhaps the best fit line could be a hierarchical model with random slopes (by country)

**Discussion**

- In the first paragraph of the Discussion, why do the findings suggest infections every few years? This is not clear from the results. Can the authors provide further comment on the duration of waning immunity from the model? Does the model extend past the follow-up visits to measure this?
- Can the authors provide more discussion about the differences between antigen targets? And perhaps recommend one to use?
- Can the authors provide further implications from this study? Do the findings suggest a recommendation to revisit the guidelines for which age groups to vaccinate? Can the findings be generalized to other settings or diseases? Are there any implications for decision making or typhoid control and prevention?

**Minor comments:**

- Spell out acronyms the first time they are used: ELISA, EDTA, 95% CrI, IQR.
- Diagnostic “test” instead of diagnostic.
- Blood culture “test” or “collection” instead of blood culture.
- Consistency with spacing between text and citations.
- Suggest using the word “affordable” instead of “cheap” diagnostic in the second paragraph of the introduction.
- No need to italicize citations of authors (i.e., Aiemjoy et al.). Also, make sure that there is a period at the end of “et al.” in every instance.
- Spell out single-digit numbers (optional).
- Please check whether “Moss.Inc” is the correct spelling and punctuation.
- In the “Ethics” section, no need to capitalize “Individual.”
