# Supplementary material for: Leveraging paired serology to estimate the incidence of typhoidal Salmonella infection in the STRATAA study
Source: PLoS Negl Trop Dis. 2025 Oct 9;19(10):e0013612. doi: 10.1371/journal.pntd.0013612 (PMC12527133; doi:10.1371/journal.pntd.0013612)
Supplement: Attachment — Submitted filename: Response_To_Reviewers.docx [file pntd.0013612.s020.docx]

We appreciate the time, effort, and thoughtful feedback that that the reviewers and editors have devoted to our manuscript. We have included a point-by-point responses to each of their comments below.

**Methods**

***Reviewer 1: Were paired samples from the same individuals run at the same time (eg on the same plate) on the Luminex?***

Paired samples from the same participant (a baseline sample and a follow-up sample) were always tested on the same plate against all antigen targets during the Luminex runs. We have revised the “*Sample collection and laboratory testing*” section of the methods to make this clearer.

Page 10, lines 6-7:

*“To ensure comparability, paired samples from the same participant were always tested on the same plate using the same set of antigen batches.”*

***Reviewer 1: For the sensitivity analysis that evaluated the impact of the time between visits, did the authors also consider an additional sensitivity analysis that removed the restriction on time between visits? Or at least examine the distribution of change in standardized IgG response in the <150d vs >=150days groups? This would be mainly relevant in Malawi which has the largest data loss due to this restriction. Given that you are examining IgG responses, these may persist at elevated levels for long periods of time, especially in highly endemic settings with repeated exposures.***

Thank you for this suggestion. We have added an additional sensitivity analysis in which all participants are included when calculating seroincidence, regardless of the elapsed time between samples. These new estimates have been added to Figure S14, alongside the seroincidence estimates based on participants with ≤150 or ≤100 days between samples.

This change had very little impact on seroincidence at the Bangladesh and Nepal study sites, since there were very few participants with >150 days between samples at these locations. As a result, including these participants increased the sample size by <1% (Bangladesh: 6,852 + 62 🡪 6,914 participants, +0.9%, Nepal: 2,549 + 1 🡪 2,550 participants, +0.04%).

At the Malawi study site, seroincidence estimates marginally decreased for all age groups and antigen targets when the 470 participants with >150 days between samples were included in the analysis (3,653 + 470 🡪 4,123 participants, +13%). This reduction in incidence is likely explained by participants who were infected and had antibody levels rise and fall during the longer interval between samples (i.e. false negatives). Nevertheless, the association between age and seroincidence was essentially unchanged in this sensitivity analysis.

We have revised the “*Data cleaning and processing*” section of the Methods and the seventh paragraph of the Results to incorporate this sensitivity analysis.

Page 10, line 22 to Page 11, line 4:

*“To minimize the impact of false-negative infections, which could occur if antibodies have enough time to undergo significant waning by the time the second sample is collected, we excluded participants with over 150 days between samples; we conducted a sensitivity analysis in which this threshold was not applied, and another in which it was reduced to 100 days. The distribution of days between samples for each study site is shown in Figure S3.”*

Page 19, lines 9-16:

*“Reducing the upper limit on time between samples from 150 to 100 days did not have a large or consistent impact on seroincidence estimates (Figure S14). Removing this limit and including participants with over 150 days between samples had very little impact on seroincidence at the Bangladesh and Nepal study sites, since few participants were excluded by this criterium (Figure S3). At the Malawi site, seroincidence estimates marginally decreased across age groups and antigen targets when the 470 participants with >150 days between samples were included in the analysis, although the relationship between age and seroincidence was essentially unchanged.”*

***Reviewer 1: Were any of the serosurvey participants among the blood-culture confirmed cases and/or the healthcare-seeking survey? Did the serosurvey include any data capture on recent history of febrile symptoms?***

Participants in the serosurvey were not asked about their symptom history, and there were no serosurvey participants at any of the study sites who also participated in the healthcare utilization survey. Given the months between samples, the potential for recall bias, and the many possible causes of febrile illness at the study sites, we believe it is unlikely that a strong association between fever symptoms and any specific seroresponse would be observed.

We have added a description of blood-culture testing results in seroserosurvey participants to the second paragraph of the Results, along with a new Figure S7.

Page 16, lines 5-15:

*“There were 47 participants who received a blood-culture test from a STRATAA study clinic during the period between the collection of the first and second serologic samples (37 from Bangladesh, 10 from Nepal, none from Malawi) (Figure S7). Four participants were blood-culture positive for either S. Typhi (n=3) or S. Paratyphi A (n=1) and experienced a 4.0-fold median rise in anti-HlyE IgG (IQR: 3.35-fold to 4.60-fold). One blood-culture-negative participant at the Bangladesh site had a positive stool-culture for S. Typhi, and experienced a 4.76-fold rise in HlyE IgG. All five of these participants were classified as infected by the HlyE mixture model. The 42 participants without a positive S. Typhi/Paratyphi culture test experienced only a 1.01-fold median change in anti-HlyE IgG (IQR: 0.89-fold to 1.10-fold), although 7 of these culture-negative participants were classified as infected by the HlyE mixture model.”*

***Reviewer 3:  In the first paragraph of the methods (Study design and enrollment of participants), were there no census updates in Malawi? To clarify, there were baseline censuses and final censuses at each site, and Nepal and Bangladesh had additional census updates in between? Please make this clearer.***

Correct, no census update was performed at the Malawi site. We have revised the text in the “Study design and enrollment of participants” section to make this clearer.

Page 8, lines 6-9:

*“The baseline population of each site’s demarcated study area was enumerated in a household census conducted between June and October 2016(4). A final census was performed at each site two years later, with two intermediate census updates at the Bangladesh site and a single update at the Nepal site. No census update was performed at the Malawi site.”*

***Reviewer 3: In “Sample collection and laboratory testing,” please give the proportion of people who provided consent (or not) and who could be located (or not). Also, please provide the percentage of children younger than 6 months old who were included. Can you provide the distribution of ages sampled? Was this age group oversampled? Was sampling done in proportion to age distribution of the population? Also, please rephrase “participants who could be located.”***

Unfortunately, we do not have information on individuals who were approached for the serosurvey enrollment but did not provide consent or could not be located at the initial visit.

The number of participants <6m old has been added to Table 1, where the age distribution of serosurvey participants can also be found. To ensure a large enough sample size to reliably estimate seroincidence, the 0-4 and 5-9 year-old age groups were oversampled relative to their share of the census population at each study site (see the newly added Figure S1). Very young children would not have a chance to be included in the random sampling frame for the serosurvey if they were born after the baseline census visit. To compensate for this, whenever a new participant was enrolled in the serosurvey, field workers also attempted to enroll any children <6 months old from the same household. However, only 18 of the 13,504 qualifying participants across the three study sites were <6 months old at the time of enrollment (16 from Bangladesh, 2 from Malawi, and none from Nepal).

We have moved all descriptions of the sampling and enrollment process in the “Sample collection and laboratory testing” section to the “Study design and enrollment of participants” section and have revised the text for clarity. We have also changed some instances of “participant” to “individual,” “patient,” or “resident” throughout the paper, given the context, to improve clarity and ensure that “participant” only refers to those who were specifically enrolled in the serosurvey.

Page 8, lines 11-21:

*“At each site, the baseline census was used to randomly select residents for the serologic survey within five age groups: 0-4, 5-9, 10-14, 15-29, and 30+ years old. The 0-4 and 5-9 year-old age groups were oversampled, relative to their share of the census population, to ensure that there would be enough participants to reliably estimate seroincidence in these age groups (Figure S1)(31). Each sampled resident was randomly assigned to a 3-month window within the year for sampling to ensure representative coverage over the study period. Field workers visited these residents at their home during this window to enroll participants and collect baseline blood samples. If the sampled resident was not available, the field workers attempted to enroll a different household member from the same age group. During this visit, field workers also attempted to enroll household members under 6 months of age bolster the number of participants from this age group.”*

***Reviewer 3: Please provide justification for choosing to use the mixture model to address the study question. Why use this model over, for example, an exponential decay model?***

The mixture model provides a flexible approach to defining seroconversion when we have paired samples, whereas an exponential decay model is more appropriate for analyzing cross-sectional data, but requires additional assumptions about the antibody kinetics. We have added the following justification to the end of the first paragraph of the “Classifying seroresponses” section of the methods.

Page 12, lines 4-7:

*“This mixture model approach takes advantage of the paired structure of the STRATAA serosurvey data, puts precise bounds on the time period during which exposure coul have occurred, and does not require us to make specific assumptions about antibody kinetic parameters, the duration of post-infection immunity, or a seropositivity threshold.”*

***Reviewer 3: There are often issues with convergence in mixture models. Please provide further model diagnostics for convergence past visual inspection.***

To test the convergence of our mixture models, we refit each model 5 times under different starting conditions. For each refitted mixture model, the number of participants classified as infected was within 2 of the model used in the main analysis, a <0.15% difference. As a result, we are confident that the mixture models are well converged. We have added this information to the second paragraph of the “Classifying seroresponses” section of the methods.

Page 12, lines 12-14:

*“To test the convergence of our mixture models, we refit each model five times under different starting conditions. For each refitted mixture model, the number of participants classified as infected was within 2 of the model used in the main analysis (<0.15% of participants).”*

***Reviewer 3: Why did the authors use the Metropolis algorithm over Metropolis-Hastings?***

In the original Metropolis algorithm, the proposal distribution for the next sample is symmetrical around the value of the current sample. The Metropolis-Hastings algorithm is a generalization of the Metropolis algorithm in which the proposal distribution is not necessarily symmetrical. Since we do use a symmetrical proposal distribution, as indicated in the text (“a normal proposal distribution with a variance of 0.1 infections per person-year”), both “Metropolis” and “Metropolis-Hastings” are technically correct names for the algorithm we use, with the former being more specific. However, since the latter name is more well known, and we explicitly describe our symmetrical proposal distribution in the text, we have changed the name from “Metropolis” to “Metropolis-Hastings” to avoid any confusion.

***Reviewer 3: Please cite R in the text (last paragraph before “Ethics”).***

We have added the following citation:

*R Core Team. R: A language and environment for statistical computing. Vienna, Austria: R Foundation for Statistical Computing; 2020. Available from: https://www.r-project.org/*

**Results**

***Reviewer 1: The Methods section describes that particular efforts were made to recruit children <6m into the study -- as such it would be helpful to know how many were included from this age group. Did the authors consider that maternal IgG may have influenced the ability to detect meaningful changes in IgG response and/or offered protection during this early period?***

Of the 13,504 participants across the three study sites who met the inclusion criteria, only 18 were less than 6 months old at the time the first sample was collected: 16 from the Bangladesh site, 2 from the Malawi site, and none from the Nepal site. We have revised Table 1 to include this information.

It’s possible that participants who were enrolled at such a young age could have received some protection against infection from maternal antibodies for at least part of the period between samples. However, since so few participants in our study were <6m old at the time of enrollment, we were not able to robustly estimate seroincidence in this age group. Excluding <6m old participants from the analysis resulted in similar or slighter higher HlyE seroincidence estimates (see the newly added Figure S8). Thus, our main observation remains unchanged: HlyE seroincidence is highest in young children, and was particularly high in the first two years of life at the Bangladesh and Malawi study sites. We have revised the fourth paragraph of the Results and the fourth paragraph of the Discussion to note this.

Page 17, lines 10-13:

*“Excluding participants who were younger than 6 months old at enrollment (16 at the Bangladesh site, two at the Malawi site, and none at the Nepal site) resulted in similar or slightly higher HlyE seroincidence estimates (Figure S8).”*

Page 24, lines 9-11:

*“Fifth, very few participants under 6 months of age were enrolled in our study, despite efforts to recruit from this population. Thus, we were not able to reliably evaluate the effect of maternal immunity in this specific age group.”*

***Reviewer 1: For Figure 3, it may be worthwhile to split the 0y group into <6m and >6m if you have enough sample size.***

We have chosen not to present a specific seroincidence estimate for those aged <6m at the time of enrollment, given the very small number of participants in this age group (16 in Bangladesh, 2 in Malawi, and none in Nepal). However, we have added a sensitivity analysis showing how HlyE seroincidence estimates in children change if <6m old participants are excluded, as noted above (Figure S8).

***Reviewer 1: The authors focus on significant positive changes in standardized IgG response to estimate seroincidence. Could you interpret significant negative changes as indication that the individuals were recently exposed prior to the baseline measurement (eg the lower half of the middle panels in Figure 1)?***

We suspect that this interpretation is correct. However, incorporating these “pre-baseline exposures” into our seroincidence calculation would have required us to make specific assumptions about how early an exposure could occur (relative to the collection of the first sample) and be considered sufficiently elevated at the first sample to be captured as a large fall in IgG between our paired samples. If this assumption was incorrect, our seroincidence estimates would be biased. Ultimately, we did not believe the extra information that we could gain outweighed the added complexity and potential bias.

***Reviewer 1: There is a statement that YncE was the only antigen target that had the same rank ordering of sites for seroincidence and clinical incidence. However there's statistically no significant difference between Malawi and Nepal for both YncE and Nepal serocincidence (Also for the legend for Supplementary Figure 5 please define the error bars). It is interesting to note that by HlyE, Nepal and Malawi are very similar in all age groups except for 0-4 -- this may be worthy of some discussion.***

We have revised the fifth paragraph of the Results section to point out that the credible intervals for YncE seroincidence overlap between study sites, even though the point estimates correctly capture the observed rank ordering by clinical incidence (Bangladesh > Nepal > Malawi). Note that after we revised our primary analysis to automatically classify participants as uninfected if they had a low absolute concentration of IgG at follow-up (see below), the median CdtB seroincidence estimates also follow this rank order.

Page 18, lines 7-12:

*“YncE and CdtB were the only antigen targets for which the median seroincidence estimates captured the overall rank ordering of enteric fever incidence between study sites, with the highest incidence at the Bangladesh site, the lowest incidence at the Malawi site, and intermediate incidence at the Nepal site. However, the 95% credible intervals of the age-standardized YncE and CdtB seroincidence estimates overlapped between study sites (Figure S11).”*

We have also revised the legend for Figure S11 (formerly Figure S5) to define the meaning of the error bars (95% credible intervals for the seroincidence estimates and adjusted clinical incidence estimates, and 95% confidence intervals for the unadjusted clinical incidence estimates), as we already had for the y-axis labels.

Finally, we have added a sentence to the end of the fifth paragraph in the results to make a note of how HlyE seroincidence differs with age between the Malawi and Nepal study sites.

Page 18, line 16-17:

*“While HlyE seroincidence in children was higher at the Nepal study site than at the Malawi site, seroincidence in adults was comparable between the two sites.”*

***Reviewer 1: The site name strip text is cut off in Supplementary Figure 8 (figure just needs to be slightly smaller to fit the page)***

Thank you for bringing this to our attention. This seems to have been caused by the conversion of the word document to pdf. At the request of the editors, we have removed the supplementary figures from the manuscript file and uploaded them separately, so this should not be a problem when the paper is formatted.

***Reviewer 3: In the first paragraph of the Results, please (re)state the inclusion criteria for the participants.***

We have revised the first sentence of the results section to restate our inclusion criteria for participants:

Page 15, lines 14-18:

*“The number of participants meeting the inclusion criteria (collection of both baseline and follow-up samples within 150 days of each other, a complete record of the sample collection dates, and for the Nepal site only, enrollment before December 2017 to avoid overlap with the TyVac-Nepal TCV trial) was 6,852 at the Bangladesh site, 3,653 at the Malawi site, and 2,549 at the Nepal site (Table 1).”*

***Reviewer 3: Please show the distribution of days between samples (perhaps in a graph). Are there any concerns or biases with having visits too close together? Does the fact that follow-up samples were collected slightly earlier in Bangladesh (vs. later for others) have any implications for the results?***

Thank you for this suggestion. We have added a new Figure S3 showing the distribution of days between samples for each study site, and renamed the other supplementary figures accordingly.

It’s unlikely that our results were biased in any meaningful way by participants with too few days between samples, since the number of days between samples (*d_i_*) is accounted for in the incidence rate estimation (equation on page 13). In theory, if the time between samples is very short, there might not be enough time for an infection during this time period to induce a large IgG response before the second sample is collected, preventing us from identifying these infections. Research published in [Aimejoy 2022](https://www.sciencedirect.com/science/article/pii/S2666524722001148?) suggests that IgG against HlyE and LPS antigens peak within three weeks of fever onset. In practice, the time between samples was much higher than this for the vast majority of participants in our study, with >99% of participants having at least 50 days between samples at each study site (see Figure S3). It’s also unlikely that the slightly earlier collection of follow-up samples at the Bangladesh site meaningfully biased our results, since excluding participants with >100 days between samples (which made the three study sites much more similar in terms of inter-sampling time) did not systematically change the seroincidence estimates (see Figure S14). We have added some text on these possibilities to the Discussion section.

Page 24, lines 4-9:

*“Fourth, there were minor differences in the timing of sample collection between the study sites. Follow-up samples were usually collected slightly earlier at the Bangladesh site relative to the Malawi and Nepal sites. However, in a sensitivity analysis that excluded participants with over 100 days between samples, in effect negating the difference between Bangladesh and the other two sites, we did not observe any systematic change in the seroincidence estimates.”*

***Reviewer 3: General comments for figures:***

- ***Please spell out all acronyms (IgG, CrI, PY, etc)***

We have revised the figures and their headings to spell out all acronyms. In the case of Figure 1, where spelling out “Immunoglobulin G” would make the plot too dense, we have defined this acronym in the figure description.

- ***Suggest using the word “panel” instead of subfigure***

We have made this change throughout the manuscript.

- ***Table 2: shouldn’t the adjusted numbers be CrI (not CI)?***

Thank you for pointing out this typo, we have corrected CI to CrI.

- ***Table 2: Are the ratios shown using the median estimates? Why not have uncertainty intervals for the ratios too?***

These ratios were calculated by dividing the median seroincidence estimate by the point estimate of clinical, and are intended to give a general measure of their relative incidence.

- ***Why use 95% CrIs in Table 2, but report IQR in Table 1 and the text for other estimates?***

In this paper, we use only use credible intervals to describe Bayesian posterior distributions of incidence, which are produced as model outputs. For observed empiric samples, such as observed IgG measurements, we believe that IQRs provide a more useful description of variability, particularly when the sample size is limited.

- ***In Table 3, please provide further details about the table (i.e., I think this is Pearson correlation?). Why are there columns without Nepal?***

We have revised Table 3 to clarify that the values represent the Pearson correlation between the median seroincidence estimate and the clinical enteric fever point estimate across each age group and study site. We have included columns without Nepal to demonstrate that the estimates for this site have the effect of weakening the correlation between seroincidence and clinical incidence, as explained in the fifth paragraph of the Results section.

- ***Figure 1: please label the scales in the middle column. Also, no need to capitalize X- and Y-axis (and this isn’t done in Figure 2)***

All 9 panels in Figure 1 are on the same scale, the only difference between the columns are how the points are colored. To clarify this, we have added labels to the panels in the middle and right columns. We have also un-capitalized “x-axis” and “y-axis” in the description of Figure 1, for consistency with the other figures.

- ***Figure 2: Can the authors put the legend altogether, instead separated by two panels? Can the authors also add the 95% CrIs for the adjusted incidence estimates?***

We have made these changes.

- ***Figure 3 & 4: please add labels for the row panels.***

We have made these changes.

**Conclusions**

***Reviewer 1: The results are well discussed and put into appropriate public health context. One minor point is that at in discussion of the disease burden in adults there is the statement: 'infections in this group are also less likely to result in symptomatic disease' -- it may be the case that these infections are either asymptomatic or mildly symptomatic but don't lead to care-seeking. The authors could propose a hypothetical study design that would help elucidate the differences between clinically significant disease vs exposure/infection that results in antibody responses (and potentially associated transmission) as this is a persistent question in the field.***

Thank you for this suggestion. We have revised the quoted statement to mention mildly symptomatic infections that don’t result in care-seeking.

Page 21, lines 10-13:

*“Our seroincidence results reveal that the low burden of disease in adults only can be partially explained by a reduced risk of infection, which suggest that infections in this group are more likely to be asymptomatic or only result in mild disease.”*

However, note that the adjusted clinical incidence estimates account for the lower reported rates of care-seeking for febrile illness among adults in Bangladesh and Malawi, as described in detail in [Phillips 2021](https://pmc.ncbi.nlm.nih.gov/articles/PMC9291985/).The differences and overlap between clinically apparent cases of disease, exposures that result in antibody responses, and infections that result in transmission are interesting and important. However, we believe that proposing a specific new study to tackle this question is beyond the scope of the paper and our specific results.

***Reviewer 3: In the first paragraph of the Discussion, why do the findings suggest infections every few years? This is not clear from the results. Can the authors provide further comment on the duration of waning immunity from the model? Does the model extend past the follow-up visits to measure this?***

Our mention of “infections every few years” is simply a reframing of our seroincidence estimates. For instance, a seroincidence estimate of 25 per 100 person-years is equivalent to 1 infection per 4 person-years. That is, we would expect the average person to be infected once in a 4-year period. (This is just an illustrative example, but our seroincidence estimates are of a similar magnitude or often higher.)

It is important to note that our modeling approach does not estimate or make any specific assumptions about the duration of immunity. The only assumption we make is that a significant rise in antibody titers between samples is indicative of a single infection, and we would not be able to account for multiple infections between samples (which is unlikely given the short time interval). We have added a statement to the “Classifying seroresponses” section of the Methods on this issue, alongside a broader discussion of our mixture modeling approach.

Page 12, lines 4-7:

*“This mixture model approach takes advantage of the paired structure of the STRATAA serosurvey data, puts precise bounds on the time period during which exposure coul have occurred, and does not require us to make specific assumptions about antibody kinetic parameters, the duration of post-infection immunity, or a seropositivity threshold.”*

***Reviewer 3: Can the authors provide more discussion about the differences between antigen targets? And perhaps recommend one?***

In the last paragraph of the Discussion, we have added a recommendation that future seroepidemiology studies focus on the HlyE antigen, given its strong association with clinical enteric fever at the population level (in this study) and at the individual level (in previous studies).

Page 25, lines 5-6:

*“We recommend that future serosurveillance studies focus on the HlyE antigen target, given its strong association with enteric fever at the population and individual level.”*

***Reviewer 3: Can the authors provide further broader public health implications from this study? Do the findings suggest a recommendation to revisit the guidelines for which age groups to vaccinate? Can the findings be generalized to other settings or diseases? Are there any implications for decision making or typhoid control and prevention?***

In the last paragraph of the Discussion, we have added a recommendation that future modeling studies evaluate the public health impact of vaccinating adults against typhoid fever, given the high seroincidence we observed in this study.

Page 25, lines 9-12:

*“Given the high seroincidence observed among adults, future studies should evaluate the public health impact and cost-effectiveness of vaccinating adults against typhoid fever. Even if infections in this age group are relatively unlikely to progress to clinically severe disease, there could be indirect benefits on population-level transmission.”*

**Editorial and Data Presentation Modifications**

***Reviewer 1: The last sentence of the clinical surveillance section in methods should read: Code and data ARE available…***

We have made this change.

***Reviewer 2: Some seroresponses shown in Figure 1 appear to originate from very low baseline antibody levels (log-sFI < -3) with large fold rises. Could these be artifacts of noise (i.e., small values divided by small values)? Please discuss whether a lower limit of detection was applied and whether any such responses were excluded or flagged.***

The reviewer raises a good point. For participants with very low IgG (near the limit of detection) at baseline, it’s possible that a small absolute rise in antibodies in the absence of infection (due to biological or measurement variability) could appear as a large fold-change and be mistakenly classified as an infection by the mixture model. We have revised our primary analysis to require that participants have a sufficiently high absolute concentration of IgG at the second visit (defined as a higher FI measurement than a negative sample tested on the same plate and antigen batch) in order to be classified as infected (in addition to the mixture model classification of a large relative rise in IgG).

To evaluate the effect of our stricter infection definition, we compared the new primary analysis seroincidence estimates to a sensitivity analysis in which the new criteria is not applied (our original estimates before making the change). The stricter definition resulted in lower seroincidence estimates, although the magnitude of the change varied between antigen targets (see the new Figure S15). Seroincidence based on the Vi antigen declined significantly at each of the study sites, particularly in the younger age groups, flattening the relationship between age and seroincidence. On the other hand, seroincidence estimates based on the HlyE, LPSO9, and YncE antigens displayed very little change. Aside from the Vi antigen, this reclassification did not meaningfully change the relationship between age and seroincidence. We have revised the second paragraph of the “*Classifying seroresponses*” section of the Methods and the eighth paragraph of the Results to incorporate this sensitivity analysis.

Page 11, line 20 to Page 12, line 3: *“We assigned each participant to the cluster with the higher posterior probability, and classified participants as infected if they had both a significant increase in IgG between visits (i.e., posterior probability of increase > 0.5) and a higher IgG concentration at the follow-up visit than a negative sample from the same plate, to exclude false-positive infections caused by small absolute rises in IgG around the assay limit of detection. All other participants were considered uninfected.”*

Page 19, line 18 to Page 20, line 6:

*“Classifying all participants with a large relative increase in IgG between visits as infected, regardless of the absolute IgG concentration at the second visit, increased our seroincidence estimates, although the effect varied between antigen targets (Figure S15). Seroincidence based on the Vi antigen increased significantly at each of the study sites, particularly in the younger age groups. However, estimates based on the HlyE, LPSO9, and YncE antigens were mostly unchanged. With the exception of the Vi antigen, our decision to classify* *all participants with a low absolute IgG concentration at follow-up as uninfected did not meaningfully affect the relationship between age and seroincidence. Finally, seroincidence estimates were consistently lower (but still substantially higher than enteric fever incidence) when we replaced binary infection status with the posterior probability of infection from the mixture model in the likelihood function (Figure S16).”*

***Reviewer 2: Consider reporting or analyzing the median FI rather than the mean. Median FI may be less sensitive to right-skewed distributions and outliers.***

In this context, duplicates always consisted of a pair of two FI measurements for each target antigen from each sample, so the median and mean would be equivalent. We have revised the *“Sample collection and laboratory testing”* section to make this clearer:

Page 10, lines 9-11:

*“We tested each sample in duplicate and recorded the mean and standard deviation of the two fluorescence intensity (FI) measurements (after subtracting the associated blank FI value) for each antigen target.”*

We also revised the second paragraph of the “*Data cleaning and processing”* section to improve clarity and make the language more consistent with that in the previous section.

Page 11, lines 9-12:

*“To standardize measurements between antigen batches, we took the mean FI value from the two sample duplicates, applied a log10 transformation, and calculated a z-score based on the batch-specific mean and standard deviation of the resulting log10(FI) values. We refer to the resulting output as the standardized fluorescence intensity (sFI).”*

***Reviewer 2: The Malawi serosurvey spans approximately 18 months and includes two peak transmission seasons, whereas the Bangladesh and Nepal serosurveys only capture one. Given assumptions about constant FOI, could the inclusion of two peak seasons within an 18-month period have overestimated the annual seroincidence in Malawi? I recommend conducting a sensitivity analysis restricting the Malawi sample to a 12-month window to assess whether this alters the rank ordering of seroincidence estimates between sites.***

We have added a sensitivity analysis for the Malawi site in which we restricted our estimates to participants with both baseline and follow-up visits in 2017. Consequently, estimates from this sensitivity analysis are only based on exposures and person-time that occurred within this 12-month period. Excluding participants outside of this period resulted in lower age-standardized seroincidence estimates for each target antigen, and changed the rank ordering of study sites for three of the targets (HlyE, LPSO2, and Vi). We have revised the third paragraph of the “Study design and enrollment of participants*”* section of the Methods, the fifth paragraph of the results, and Figure S11 (formerly Figure S5) to incorporate this sensitivity analysis.

Page 9, lines 7-12:

*“In addition, seroincidence estimates for Malawi could be biased due to seasonality in enteric fever incidence, since the Malawi serosurvey period was approximately 18 months and overlapped with two seasonal peaks in transmission. Therefore, we performed a sensitivity analysis for the Malawi site in which participants who did not have both samples collected during 2017 were excluded.”*

Page 18, lines 12-16:

*“Limiting our Malawi analysis to participants with both samples collected in 2017 resulted in lower seroincidence estimates for each antigen target (Figures S11-S12). As a result, the rank ordering of study sites by age-standardized seroincidence matched that of enteric fever incidence for the CdtB, HlyE, Vi, and YncE antigen targets in this sensitivity analysis.”* ***Reviewer 2: The adjustment factors applied to clinical incidence in Nepal appear unusually large in older age groups (e.g., age 10–14: from 0.28 to 5.47). Could potential issues in the adjustment methodology explain the discordance between clinical and serologic incidence in these age groups in Nepal? Please consider discussing this possibility in the limitations section.***

We agree that the large adjustment factors estimated based on the healthcare utilization survey in Nepal could make this data less reliable (which is why we also estimated the Pearson correlation coefficient between the seroincidence and observed/adjusted clinical incidence excluding Nepal in Table 3). We have added a brief discussion of this potential issue to the limitations section, in the fourth paragraph of the Discussion.

Page 24, lines 11-15:

*“Sixth, the estimated adjustment factors for enteric fever incidence were higher at the Nepal site than at the Bangladesh and Malawi study sites, particularly in the 5-9 and 10-14 year-old age groups. Overestimation of these adjustment factors at the Nepal site could have weakened the observed correlation between enteric fever incidence and seroincidence.”*

***Reviewer 2: The use of a 0.5 posterior probability threshold to classify participants as infected versus not infected could be better justified. Was this threshold chosen arbitrarily or based on prior data or validation? Did the authors consider alternative thresholds? A brief discussion of the rationale and impact on seroincidence estimates would be helpful.***

Since our mixture model contains two components (one corresponding to a large change in IgG between visits, the other corresponding to little/no change in IgG), our use of the 0.5 threshold is equivalent to assigning participants to the cluster with the highest posterior probability. As a result, using a higher or lower threshold would be expected to artificially bias our classification of infection status and therefore our seroincidence estimates. In practice, the mixture model components were well separated for each study site and antigen target, such that most participants were assigned a posterior probability close to either 0 or 1 (see the newly added Figure S4). Therefore, our seroincidence estimates should be relatively robust to the specific posterior probability cutoff used to classify infections. We have added a sentence on this to the second paragraph of the “Classifying seroresponses” section of the Methods.

Page 12, lines 10-11:

*“The full distribution of participants’ posterior probability of infection from the mixture model are given in Figure S4 for each study site and target antigen.”*

We also performed a sensitivity analysis in which we directly incorporated the posterior probabilities from the mixture model into the seroincidence likelihood function, which eliminates the need for a specific cutoff altogether (see Figure S16). Our seroincidence estimates are somewhat lower when using this alternative probabilistic likelihood function, but still much greater than clinical incidence, and the association between seroincidence and age is preserved.

***Reviewer 2:  Assay Quality Control***

- ***Please describe whether blank values were subtracted from the fluorescence intensity (FI) measurements. Were results normalized to a positive control or reference standard? If so, please describe the method.***

We subtracted blank values from the FI measurements before analyzing them, although we did not normalize them against a specific control or reference standard. In addition, paired samples from the same participant were always tested in parallel on the same plate and using the same antigen batches. We have revised the “*Sample collection and laboratory testing”* section of the Methods to make this clearer.

Page 10, lines 6-11:

*“To ensure comparability, paired samples from the same participant were always tested on the same plate using the same set of antigen batches. Each plate also included a blank control (diluent with no sera) and a negative control sample taken at baseline from a presumably unexposed participant in a typhoid human challenge study(32). We tested each sample in duplicate and recorded the mean and standard deviation of the two fluorescence intensity (FI) measurements (after subtracting the associated blank FI value) for each antigen target.”*

To control for possible differences in reactivity between antigen batches, we Z-score standardized the log10(FI) values against the batch-specific mean and standard deviation for each antigen target and study site.

- ***The manuscript mentions that results were collected as duplicate mean FI; please report the within-plate coefficient of variation (CV) across duplicates. If any samples were repeated across plates, also report the across-plate CVs.***

We have added the following information to the “*Sample collection and laboratory testing”* section of the Methods, as well as a newly added Figure S2.

Page 10, lines 9-16:

*“We tested each sample in duplicate and recorded the mean and standard deviation of the two fluorescence intensity (FI) measurement (after subtracting the associated blank FI value) for each antigen target. To quantify measurement variability, we calculated the coefficient of variation (CV) for each participant and antigen target as the ratio of the standard deviation to the mean of the duplicate FI measurements. Across participants, the median CV ranged from 1.56% (interquartile range (IQR): 0.64% to 3.43%) for HlyE at the Bangladesh study site to 9.40% (IQR: 4.07% to 18.99%) for Vi at the Nepal study site (Figure S2).”*

- ***A description of quality control procedures should be included in the main text or supplement.***

We have added a second paragraph to the “*Sample collection and laboratory testing”* section to discuss this.

Page 10, lines 6-16:

*“To ensure comparability, paired samples from the same participant were always tested on the same plate using the same set of antigen batches. Each plate also included a blank control (diluent with no sera) and a negative control sample taken at baseline from a presumably unexposed participant in a typhoid human challenge study(32). We tested each sample in duplicate and recorded the mean and standard deviation of the two fluorescence intensity (FI) measurements (after subtracting the associated blank FI value) for each antigen target. To quantify measurement variability, we calculated the coefficient of variation (CV) for each participant and antigen target as the ratio of the standard deviation to the mean of the duplicate FI measurements. Across participants, the median CV ranged from 1.56% (interquartile range (IQR): 0.64% to 3.43%) for HlyE at the Bangladesh study site to 9.40% (IQR: 4.07% to 18.99%) for Vi at the Nepal study site (Figure S2).”*

***Reviewer 2:  Consider replacing the term “reported incidence” with “clinical incidence (crude and adjusted)” throughout. "Reported" seems to imply passive surveillance, whereas these estimates come from active prospective surveillance studies.***

We have replaced “reported” with “crude” and “unadjusted” throughout the text.

***Reviewer 2:  The higher seroincidence rates for LPSO2 and O9 in Malawi may be indicative of a higher burden of non-typhoidal Salmonella at this site.***

Thank you for pointing this out. We have added a brief discussion of this possibility to the fourth paragraph of the Discussion section.

*Page 23, lines 9-13:*

*“Cross-reactivity would inflate our seroincidence estimates and weaken their association with enteric fever incidence at the population level. For instance, the relatively high LPSO2/O9 seroincidence at the Malawi study site could be partially explained by greater exposure to non-typhoidal Salmonella strains in this setting, relative to Bangladesh and Nepal(4).”*

***Reviewer 3: The final sentence of the manuscript would be strengthened by highlighting a key implication. For example: “…to reduce the burden of disease” rather than simply “to generate data.”***

We have added two additional sentences to the end of the last paragraph in the Discussion section, to highlight a key implication of our study on the design of typhoid vaccination programs.

Page 25, lines 9-12:

*“Given the high seroincidence observed among adults, future studies should evaluate the public health impact and cost-effectiveness of vaccinating adults against typhoid fever. Even if infections in this age group are relatively unlikely to progress to clinically severe disease, there could be indirect benefits on population-level transmission.”*

***Reviewer 1:  Visualization***

- ***Consider combining Figures 3 and 4 into a single figure showing seroincidence by year of age (0–4) and then by age group.***

We attempted to condense this information into a single figure, but concluded splitting it across the two figures provides the best balance of detail and visual clarity.

- ***For Supplemental Figure 1: Could you include higher resolution in the younger age range (0–4 years) as in Figure 3 to better explore potential maternal antibody effects? Alternatively, modeling antibody responses over continuous age using a generalized additive model (GAM) may provide improved resolution to detect maternal antibody decay.***

We have revised this figure (now known as Figure S5) to break the 0-4y age-group into individual years of age.

- ***For Supplemental Figure 1: Using a free y-axis may improve visibility of differences by site across antigen targets.***

We have made this change (now Figure S5).

***Reviewer 3: Suggestion for wording in paragraph 1 of introduction: “As a result, enteric fever mostly occurs in settings with limited [access to] clean water and sanitation…”***

Thank you for this suggestion; we have implemented this change.

***Suggestion for wording in paragraph 2 of introduction: “The absence of [robust and routine] surveillance data [limits evidence-based decision making regarding typhoid control and prevention without complete data]”***

We agree that this change improves the readability of the paragraph and have incorporated a modified version of it in the text.

Page 5, lines 20-22:

*“The absence of robust and routine surveillance data limits policymakers ability to make evidence-based decisions around typhoid control and prevention.”*

***Reviewer 3: The third paragraph in the introduction needs some sort of transition; as it is, it seems a little abrupt and out of the blue. It would be helpful to start with a transition sentence offering serosurveillance as a solution to the lack of routine surveillance data.***

We have added the following sentence to the beginning of the third paragraph, which we believe better connects it to the preceding paragraph on typhoid surveillance.

Page 6, lines 1-2:

*“In settings where routine clinical surveillance data is limited or unavailable, serosurveillance can provide valuable insights into local epidemiological conditions.”*

***Reviewer 3: The last paragraph of the introduction does not make the objective of the study as clear as it should be (as it is in the abstract). In that paragraph, it would be helpful to see: 1) motivation and rationale for the study, 2) (optional) brief overview of methods, and 3) and a reframing of the objective of the study. This is unclear as it currently is.***

Thank you for this suggestion. After revisiting the text, we agree that the objective of the study was not as clear in the Introduction as it was in the abstract. We have expanded the last sentence of the Introduction into a separate paragraph:

Page 7, lines 13-20:

*“In this study, we use a novel multiplex assay to measure IgG responses against seven typhoidal antigens in paired blood samples from healthy children and adults in the Bangladesh, Malawi, and Nepal STRATAA sites. We then fit mixture models to data on the change in IgG over time to identify infected participants and estimate seroincidence. Finally, we compare the resulting seroincidence estimates to enteric fever incidence. This approach allows us to overcome the limitations of Vi-based serology and provide a comprehensive overview of typhoidal seroincidence, its variation across different age groups and settings, and its relationship with clinical disease in the STRATAA study.”*

***Reviewer 3: Additional minor comments:
• Spell out acronyms the first time they are used: ELISA, EDTA, 95% CrI, IQR.
• Diagnostic “test” instead of diagnostic.
• Blood culture “test” or “collection” instead of blood culture.
• Consistency with spacing between text and citations.
• Suggest using the word “affordable” instead of “cheap” diagnostic in the second paragraph of the introduction.
• No need to italicize citations of authors (i.e., Aiemjoy et al.). Also, make sure that there is a period at the end of “et al.” in every instance.
• Spell out single-digit numbers (optional).
• Please check whether “Moss.Inc” is the correct spelling and punctuation.
• In the “Ethics” section, no need to capitalize “Individual.”***

We have implemented these changes throughout the manuscript. “Moss.Inc” was a typo, and has been corrected to “Moss Inc.”
